# A Systems Biology-Based Gene Expression Classifier of Glioblastoma Predicts Survival with Solid Tumors

**DOI:** 10.1371/journal.pone.0006274

**Published:** 2009-07-17

**Authors:** Jing Zhang, Bing Liu, Xingpeng Jiang, Huizhi Zhao, Ming Fan, Zhenjie Fan, J. Jack Lee, Tao Jiang, Tianzi Jiang, Sonya Wei Song

**Affiliations:** 1 LIAMA Center for Computational Medicine, National Laboratory of Pattern Recognition, Institute of Automation, Chinese Academy of Sciences, Beijing, the People's Republic of China; 2 Cancer Research Laboratory, Beijing Shijitan Hospital, The Capital Medical University, Beijing, the People's Republic of China; 3 Department of Biostatistics, The University of Texas M. D. Anderson Cancer Center, Houston, Texas, United States of America; 4 Glioma Therapy Center, Beijing Tiantan Hospital, Beijing, the People's Republic of China; Center for Genomic Regulation, Spain

## Abstract

Accurate prediction of survival of cancer patients is still a key open problem in clinical research. Recently, many large-scale gene expression clusterings have identified sets of genes reportedly predictive of prognosis; however, those gene sets shared few genes in common and were poorly validated using independent data. We have developed a systems biology-based approach by using either combined gene sets and the protein interaction network (Method A) or the protein network alone (Method B) to identify common prognostic genes based on microarray gene expression data of glioblastoma multiforme and compared with differential gene expression clustering (Method C). Validations of prediction performance show that the 23-prognostic gene classifier identified by Method A outperforms other gene classifiers identified by Methods B and C or previously reported for gliomas on 17 of 20 independent sample cohorts across five tumor types. We also find that among the 23 genes are 21 related to cellular proliferation and two related to response to stress/immune response. We further find that the increased expression of the 21 genes and the decreased expression of the other two genes are associated with poorer survival, which is supportive with the notion that cellular proliferation and immune response contribute to a significant portion of predictive power of prognostic classifiers. Our results demonstrate that the systems biology-based approach enables to identify common survival-associated genes.

## Introduction

The prediction of survival is critical when formulating a proper treatment strategy for a patient with cancer. Clinicopathological factors such as age, sex, and tumor grade are commonly used to assess prognosis, but the prediction is limited. In glioma, tumor grade is the most established predictor of disease outcome [1], but patients with either grade III glioma or glioblastoma multiforme (GBM), grade IV glioma, are nearly uniformly fatal [2]–[4]. The subtle distinction of these two grades often misclassifies them into either grade III glioma or GBM. Even histologically identical tumors can behave in highly different manners from treatment response to survival. Patients with GBM show remarkable variations in survival from less than one week to more than three years following diagnosis [5]. Thus, new prognostic classifiers are urgently needed to more accurately predict the survival of individual patients.

Microarray gene expression signatures have been reported to predict survival of cancer patients [6]. However, sets of predictive genes generated with the differential expression clustering share few overlapping genes and exhibit less successful predictive power in independent data [6]. The lack of agreement in prediction raised doubts about the reliability and robustness of the reportedly predictive genes. There are three major causes for the divergent results: small groups of samples, complex nature of high-throughput microarray technologies, and simplified analytical methods in microarray data analysis. The use of small samples in expression profiling makes it difficult to identify genes associated with a condition or outcome, such as survival, from hundreds or even thousands of genes that exhibit expression changes [6]. Highly variable microarray experimental conditions and the use of different microarray platforms cause poor reproducibility of microarray measurements within and between laboratories [7]–[11]. Differential gene expression clustering (the SAM-based analysis) is a common analytical tool used to analyze microarray data. However, this method bases on only differential expression of individual genes for target gene identification and ignores prior knowledge of biological pathways that are composed of groups of genes and interactions of their proteins, which is believed to be more informative than expression changes of individual genes [7].

Several systematic approaches have been recently proposed to address the problems. One approach uses a gene pathway-based analysis, which identifies biological pathways (*a priori* defined gene sets) by scoring the coherency of expression changes among their member genes based on microarray data [7]–[9]. A gene set is an a priori defined set of genes, in which genes share a similar biological function or belong to one gene signaling pathway. Such a method allows biologists to incorporate previously accumulated biological knowledge in the analysis and make a more biology-driven analysis of microarray data, which can lead to identify interpretable discriminative signature that gains insights into tumor biology and potential therapeutic targets. In addition, this method enables to identify moderately differentially expressed but functionally important genes, which are missed in gene expression clustering [8]. The method has been applied to discriminate irradiated from non-irradiated yeast cells [9]. Another approach is a protein interaction network-based method, which utilizes a recently available protein-protein interaction network to identify sub-networks based on coherent expression patterns of their genes [12], [13]. A sub-network refers to a smaller or more focused network within a large protein interaction network. It has been applied to effectively differentiate a metastatic from a non-metastatic breast tumor [10]. Both the methods efficiently utilize co-expression information embedded within the microarray gene expression data. However, the problem of both the methods is that each gene set or sub-network identified includes too many genes (∼tens of genes each), which greatly limits their clinical application.

Here, we develop a systems biology-based approach (Method A) by combining gene sets and the protein interaction network to identify prognostic genes using microarray expression data of primary GBMs and compare with other two methods, Method B, which uses the protein interaction network alone, and Method C, which is based on differential gene expression patterns. We find that the 23-prognostic gene classifier identified by Method A predicts not only the survival of glioma patients, but also the survival of patients with other solid tumors such as breast cancer, lung cancer, bladder cancer, and ovarian cancer, with the success on 17 of 20 independent sample cohorts compared with 5–9 of 20 performed by the classifiers identified by other two methods. We find that the 23-gene prediction is independent of tumor grade and patient age, and 21 of the 23 genes are associated with cell proliferation while the other 2 genes associated with immune response, supporting the notion that cell proliferation and immune response exhibit a significant prognostic power. Our findings suggest that the 23-gene classifier may have general utility in predicting survival for solid tumors.

## Results

### Prognostic Classifier Development Using Systematic and Gene Expression Clustering Approaches for Primary GBM

**Figure 1:**
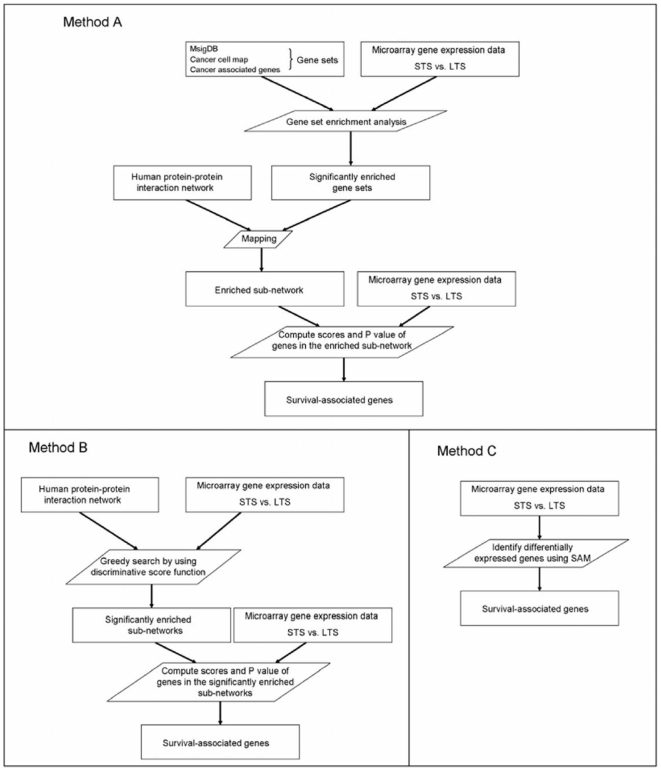
Flowchart of Methods A, B and C. Schematic method overview of a systems biology-based approach using either combined gene sets and the protein interaction network (Method A) or the protein interaction network alone (Method B) and conventional gene expression clustering (the SAM-based analysis) (Method C) for prognostic gene identification based on microarray gene expression data of primary GBMs.

To sufficiently utilize microarray expression data and the recently available protein interaction network, we developed a systems biology-based approach to identify prognostic gene classifiers based on microarray expression data of primary GBMs by using either combined gene sets and the protein interaction network (Method A) or the protein interaction network alone (Method B), and compared with conventional gene expression clustering (the SAM-based analysis) (Method C) for prognostic gene identification. The overall method strategy was outlined in Figure 1 and Materials and methods.

We collected five advanced glioma data sets, two GBM sets from the cohorts UCSF-1 (MD Anderson cancer center database) [14] and UCSF-2 (Stanford microarray database) [15] and three HGG (grade III and GBM combined) sets from the cohorts UCLA (GEO GDS1975) [3], MDA (GEO GDS1815) [4], and CMBC (BROAD institute database) [2] (Table 1). Among the five cohorts, UCLA, UCSF-1 and MDA have 35, 34, and 49 primary GBMs respectively, while CMBC and USCF-2 have only 14 and 15 primary GBMs, separately. Therefore, we used the three larger primary GBM sets (UCSF-1, UCLA, and MDA) to train a molecular classifier and left out the rest two GBM sets from UCSF-2 and CMBC, and the three HGG cohorts for validation. Considering experimental variations, different microarray platforms used, and diverse patient populations existed among the three training cohorts, we decided to apply the method (A, B, and C) to each cohort independently and then reconciled candidate prognostic genes from the three data sets to derive a final list of prognostic genes based on their overlaps between any two cohorts and concordant expression across the three cohorts with significant expression in at least two cohorts.

**Table 1:** Summary statistics of cohort data.

Tumor type	Cohort (Ref)	Platform	Median OS(m)	Age mean (sd) (yr)	Outcome	Tumor grade/stage (sample size)
**Training cohort**						
Primary GBM	UCLA [3]	Oligos Affymetrix	14	54 (15)	22^a^	IV (35)^c^
	MDA [4]	Oligos Affymetrix	17.5	49 (12)	46^a^	IV (49)^c^
	UCSF-1 [14]	Oligos Affymetrix	17	NA	34^a^	IV (34)^c^
**Validation cohort**						
GBM	CMBC [2]	Oligos Affymetrix	12	NA	24^a^	IV (28)^c^
	UCSF-2 [15]	Spotted cDNA	10	62 (21)	15^a^	IV (20)^c^
HGG (Grade III+GBM)	UCLA [3]	Oligos Affymetrix	15	46 (16)	48^a^	III (24); IV (50)^c^
	MDA [4]	Oligos Affymetrix	23	46 (13)	62^a^	III (21); IV (55)^c^
	CMBC [2]	Oligos Affymetrix	17	NA	33^a^	III (22); IV (28)^c^
Breast	GIS [16]	Oligos Affymetrix	122	65 (14)	54^a^	I (62); II (121); III (51)^c^
	CRCM [17]	Spotted Oligos	66	68 (10)	38^a^	I (21); II (94); III (33)^c^
	SUSM [18]	Oligos Agilent	87	44 (6)	79^a^	I (75); II (101); III (119)^c^
	NCI [19]	Oligos Rosetta inkjet	65	NA	34^b^	I (78)^c^
	EMC [20]	Oligos Affymetrix	86	NA	107^b^	I+II+III (286)^c^
Lung	DFCI [21]	Oligos Affymetrix	50	64 (11)	31^a^	I (49); II (13)^d^
	PCH [22]	Spotted Oligos	53	63 (10)	23^a^	I (48)^d^
	CAN/DF [23]	Oligos Affymetrix	40	61 (10)	28^a^	I (56); II (26)^d^
	MSK [23]	Oligos Affymetrix	47	67 (9)	23^a^	I (63); II (20)^d^
	UM-HLM [23]	Oligos Affymetrix	56	66 (10)	118^a^	I (160); II (48)^d^
Bladder	AUH [24]	Oligos Affymetrix	13	NA	30^a^	III+IV (30)^c^
Ovarium	MNI(GSE8842)	Spotted cDNA	80	52 (12)	13^a^	I (68)^d^

a Death.

b Metastasis.

c Tumor grade.

d Tumor stage. NA, not available. m, month. Yr, year. Ref, reference.

**Figure 2:**
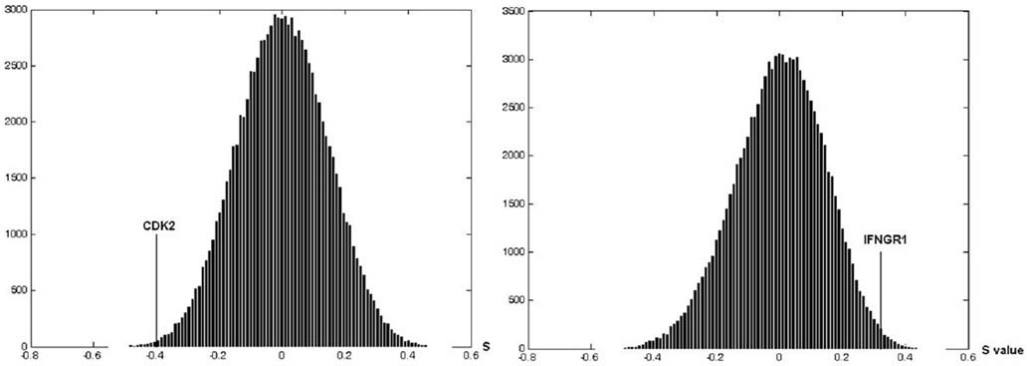
The null distributions of S values of CDK2 and IFNGR1. The null distribution of S values of CDK2 and IFNGR1 are computed from MDA-GBM data. The left panel is the null distribution of S value of cell proliferation-related gene CDK2; the right panel is the null distribution of S value of immune response-related gene IFNGR1.

Using the median OS as a cutoff for each cohort, we divided microarray data of patients into short-term versus long-term survival groups. By applying Method A, we identified 124, 114, and 78 significantly enriched gene sets between the two survival groups for UCSF-1, MDA, and UCLA, respectively (Tables S1, S2, S3). From those enriched gene sets, 198, 257, and 164 candidate prognostic genes were identified for the three cohorts, respectively, (for candidate prognostic genes and their scores, please see Tables S4, S5, S6). Based on overlap between any two cohorts and concordant expression across the three cohorts with significant differential expression (*P*<0.05) in at least two cohorts, we identified 23 prognostic genes (Table 2), from which “cyclin-dependent kinase 2” (CDK2) and “interferon gamma receptor 1” (IFNGR1) were selected to demonstrate the null distribution of their S values (Figure 2).

**Table 2:** List of prognostic genes developed by method A from primary GBM data of UCLA, USCF-1, and MDA.

Description	Gene Symbol	Entrez ID
minichromosome maintenance deficient 6	MCM6	4175
gamma 1	TUBG1	7283
thymidylate synthetase	TYMS	7298
cyclin B1	CCNB1	891
WEE1 homolog (S. pombe)	WEE1	7465
cyclin A2	CCNA2	890
CDC28 protein kinase regulatory subunit 1B	CKS1B	1163
cyclin B2	CCNB2	9133
cell division cycle 7 homolog	CDC7	8317
uracil-DNA glycosylase	UNG	7374
replication protein A3, 14 kDa	RPA3	6119
CDC6 cell division cycle 6 homolog (S. cerevisiae)	CDC6	990
CDC45 cell division cycle 45-like (S. cerevisiae)	CDC45L	8318
cyclin-dependent kinase 2	CDK2	1017
replication protein A2, 32 kDa	RPA2	6118
ribonucleotide reductase M1 polypeptide	RRM1	6240
DNA (cytosine-5-)-methyltransferase 1	DNMT1	1786
proliferating cell nuclear antigen	PCNA	5111
ribonucleotide reductase M2 polypeptide	RRM2	6241
pituitary tumor-transforming 1	PTTG1	9232
gamma-glutamyl hydrolase	GGH	8836
ras-related C3 botulinum toxin substrate 2	RAC2	5880
interferon gamma receptor 1	IFNGR1	3459

By using Method B, We obtained 147, 278, and 162 significantly enriched sub-networks between the two survival groups for UCSF-1, UCLA, and MDA, respectively. From those enriched sub-networks, we found 139, 131, and 133 candidate prognostic genes for UCSF-1, MDA, and UCLA, respectively, (for candidate prognostic genes and their scores, please see Tables S7, S8, S9). Similarly, we identified 6 prognostic genes overlapped between any two cohorts and concordantly expressed across the three cohorts with significant differential expression (*P*<0.05) in at least two cohorts (Table S10).

In Method C, we performed two-class analysis (students' t test) of microarray gene expression data to identify genes associated with survival using the SAM software. We selected the same number of top discriminative genes as the number of genes identified by Method A for UCLA, UCSF-1, and MDA cohorts, respectively, (Table S11, S12, S13). Similarly, we identified 11 prognostic genes overlapped between any two cohorts and concordant expression across the three cohorts with significant differential expression (*P*<0.05) in at least two cohorts (Table S14).

### Validation of Classifier Performance in Advanced Gliomas

To compare prediction performance of the three prognostic gene classifiers identified by Method A, B and C, we validated them in the three training primary GBM cohorts (UCSF-1, MDA, and UCLA), two independent GBM cohorts (CMBC and USCF-2), and three HGG cohorts (UCLA, MDA, and CMBC). As shown in Table 3, the multivariate Cox regression analysis indicated that the 23-gene classifier found by Method A was independently and significantly associated with survival in six of eight cohorts and moderately in CMBC-HGG (HR = 2.11; 95% CI, 1.03–4.35; *P* = 0.055), but not significantly in the training set UCLA. Similarly, the 6-gene classifier by Method B had a significant association with survival in six of the eight cohorts, but failed in the training cohort UCLA and the validation cohort UCLA-HGG. However, the 11-gene classifier by Method C was the worst predictor, which failed in four of the eight cohorts, the training cohort UCLA and three validation cohorts CMBC-GBM, UCLA-HGG, and MDA-HGG. While all the three gene classifiers were not predictive in the training cohort UCLA, the 23-gene signature seemed to have the best performance across all the cohorts assessed, followed by the 6-gene signature and then the 11-gene signature in advanced gliomas.

**Table 3:** Multivariate Cox regression analysis in training and validation cohorts of advanced gliomas.

Glioma	Cohort	Covariate	23 genes^a^		6 genes^b^		11 genes^c^	
			P value	HR (CI95%)	P value	HR (CI95%)	P value	HR (CI95%)
**Training cohort**								
GBM	UCLA	cluster	0.131	1.96 (0.82–4.71)	0.095	2.48 (0.85–7.20)	0.590	1.30 (0.50–3.42)
		age	0.001	1.06 (1.03–1.10)	0.007	1.05 (1.01–1.09)	0.001	1.06 (1.02–1.10)
	UCSF-1	cluster	0.005	3.19 (1.43–7.13)	0.001	4.22 (1.88–9.45)	0.005	2.95 (1.39–6.27)
	MDA	cluster	0.013	2.29 (1.19–4.41)	0.034	1.94 (1.05–3.59)	<0.0001	3.16 (1.66–6.01)
		age	0.522	1.01 (0.98–1.04)	0.905	1.00 (0.97–1.03)	0.761	1.01 (0.97–1.02)
**Validation cohort**								
GBM	CMBC	cluster	0.012	3.05 (1.27–7.30)	0.03	2.73 (1.11–6.76)	0.074	2.37 (0.92,6.12)
	UCSF-2	cluster	0.037	4.24 (1.09–16.52)	0.045	3.77 (1.03–13.83)	0.04	4.05 (1.21–13.49)
		age	0.09	1.04 (0.99–1.09)	0.069	1.05 (1.00–1.10)	0.055	1.05 (1.00–1.11)
HGG (Grade III+GBM)	UCLA	cluster	0.037	1.94 (1.04–3.60)	0.417	1.30 (0.70–2.43)	0.879	1.05 (0.54–2.04)
		grade	0.011	2.84 (1.27–6.35)	0.003	3.27 (1.48–7.20)	0.004	3.31 (1.46–7.05)
		age	0.217	1.01 (0.99–1.04)	0.209	1.01 (0.99–1.04)	0.151	1.02 (0.99–1.04)
	MDA	cluster	0.005	2.09 (1.25–3.49)	0.014	1.96 (1.15–3.34)	0.268	1.49 (0.74–3.00)
		grade	0.015	2.54 (1.20–5.38)	0.01	2.63 (1.26–5.06)	0.077	2.14 (0.92–4.95)
		age	0.142	1.02 (0.99–1.04)	0.184	1.02 (0.99–1.04)	0.248	1.01 (0.99–1.04)
	CMBC	cluster	0.055	2.11 (1.03–4.35)	0.001	2.84 (1.34–5.98)	0.028	2.71 (1.11–6.57)
		grade	0.01	2.66 (1.26–5.63)	0.045	2.20 (1.02–4.74)	0.36	1.54 (0.61–3.91)

* The direction of the hazard ratio is as follows: cluster, the short-term versus long-term survival group; grade, GBM versus grade III; age, older versus younger.

a Method A.

b Method B.

c Method C.

**Figure 3:**
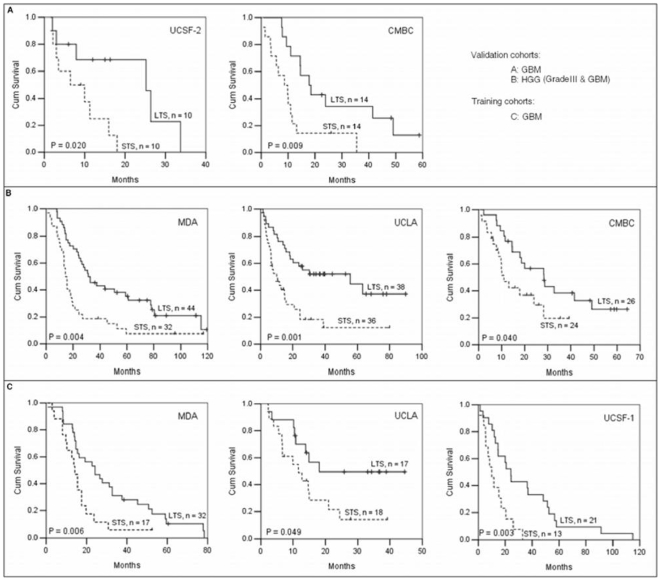
Kaplan-Meier plots of overall survival for advanced gliomas generated by the 23-gene classifier. (A) Two GBM cohorts UCSF-2 and CMBC. (B) Three HGG cohorts MDA, UCLA, and CMBC. (C) Three GBM training cohorts MDA, UCLA, and UCSF-1. STS, short-term survival group; LTS, long-term survival group. N, the number of patients within STS or LTS group. *P* values are indicated within plots. *P*< = 0.05 is defined as significance.

Then, we compared the classification power of the three gene classifiers. We generated Kaplan-Meier plots to illustrate survival differences in the two survival groups for both the validation and the training cohorts (Figure 3). The 23-gene classifier significantly classified patients into two groups with distinctively different survival time (Figure 3). Kaplan-Meier plots of the 6-gene and 11-gene classifiers on all the cohorts were present in Figure S1, S2, S3, S4, S5, S6. Alike the 23-gene classifier, the 6-gene classifier significantly discriminated patients into two survival groups in all the cohorts assessed, while the 11-gene classifier did not work well in two GBM cohorts (UCLA and CMBC). The results indicate that the 23-gene and 6-gene signatures are predictive of survival equally well on all the eight advanced glioma cohorts, better than the 11-gene signature.

### Validation of Classifier Performance in Other Tumor Types

To assess the robustness of survival prediction of the three gene classifiers for patients with other tumor types, we obtained 12 cohorts including five breast cancer cohorts: GIS (ArrayExpress E-GEOD-3494) [16], CRCM (GEO GSE9893) [17], SUSM (Stanford microarray database) [18], NCI (Rosetta inpharmatics inc database) [19], EMC (GEO GSE2034) [20], five lung cancer cohorts: DFCI (BROAD institute database) [21], PCH (GEO GSE5843) [22], CAN/DF (caArray) [23], MSK (caArray) [23], UM-HLM (caArray) [23], one bladder cancer cohort AUH (GEO GSE5287) [24], and one ovarian cancer cohort MNI (GEO GSE8842) with microarray expression data and clinicopathogical information publicly available (detailed in Materials and methods) (Table 1). According to the multivariate Cox regression analysis (Table 4), we found that the 23-gene classifier achieved an independent and significant association with survival in nine of the 12 cohorts, moderate in the cohort MSK (lung cancer) (HR = 2.29; 95% CI, 0.98–5.33; *P* = 0.056), but not significant in the two cohorts SUSM (breast cancer) and UM-HLM (lung cancer). In contrast, the 6-gene and 11-gene classifiers were very poorly associated with survival in those cohorts. They failed in ten and nine of the 12 cohorts, respectively, and were only significantly associated with survival in two cohorts: EMC (breast cancer) and MNI (ovarian cancer), and three cohorts: CRCM (breast cancer), EMC (breast cancer), and AUH (bladder cancer), respectively. Altogether, the findings demonstrate that the 23-gene classifier outperforms the other two gene classifiers in those tumor types, supporting its validity of prognosis independent of tumor grade or stage and patient age across solid tumor types.

**Table 4:** Multivariate Cox regression analysis in validation cohorts of other tumor types.

Tumor type	Cohort	Covariate	23 genes^a^		6 genes^b^		11 genes^c^	
			P value	HR (CI 95%)	P value	HR (CI 95%)	P value	HR (CI 95%)
Breast	GIS	cluster	0.003	2.37 (1.34–4.18)	0.267	1.41 (0.77–2.58)	0.42	1.27 (0.71–2.28)
		grade	0.015	1.69 (1.11–2.57)	0.003	1.89 (1.24–2.88)	0.002	1.93 (1.27–2.95)
		age	0.758	1.00 (0.98–1.02)	0.887	1.00 (0.98–1.02)	0.808	1.00 (0.98–1.02)
	CRCM	cluster	<0.0001	5.51 (2.66–11.41)	0.079	1.82 (0.93–3.54)	<0.0001	5.10 (2.46–10.57)
		grade	<0.0001	4.41 (2.37–8.22)	<0.0001	3.27 (1.85–5.81)	<0.0001	4.18 (2.27–7.71)
		age	0.688	0.99 (0.96–1.03)	0.757	1.00 (0.96–1.03)	0.751	0.99 (0.96–1.03)
	SUSM	cluster	0.08	1.60 (0.95–2.69)	0.396	1.26 (0.76–2.07)	0.955	1.01 (0.65–1.58)
		grade	0.001	2.35 (1.63–3.40)	<0.0001	2.56 (1.80–3.65)	<0.0001	2.59 (1.82–3.68)
		age	0.03	0.96 (0.92–1.00)	0.034	0.96 (0.92–1.00)	0.024	0.96 (0.92–0.99)
	NCI	cluster	0.014	4.40 (1.34–14.41)	0.324	1.41 (0.71–2.77)	0.336	1.42 (0.69–2.92)
	EMC	cluster	0.006	1.71 (1.17–2.51)	0.022	1.59 (1.07–2.37)	0.004	1.74 (1.19–2.56)
Lung	DFCI	cluster	0.012	2.53 (1.23–5.24)	0.299	1.48 (0.71–3.07)	0.162	1.71 (0.81–3.62)
		stage	0.167	1.76 (0.79–3.91)	0.129	1.86 (0.84–4.14)	0.211	1.69 (0.74–3.84)
		age	0.091	1.03 (1.00–1.07)	0.125	1.03 (0.99–1.08)	0.154	1.03 (0.99–1.07)
	PCH	cluster	0.039	2.44 (1.04–5.68)	0.15	1.85 (0.80–4.27)	0.145	1.89 (0.80–4.45)
		age	0.216	1.03 (1.00–1.07)	0.288	1.02 (0.98–1.07)	0.202	1.03 (0.99–1.07)
	CAN/DF	cluster	0.044	2.08 (1.02–4.25)	0.208	1.63 (0.76–3.48)	0.775	1.11 (0.55–2.24)
		stage	0.007	2.54 (1.29–4.99)	0.016	2.42 (1.18–4.96)	0.003	2.79 (1.41–5.52)
		age	0.001	1.08 (1.04–1.14)	0.001	1.08 (1.03–1.14)	0.002	1.07 (1.03–1.12)
	MSK	cluster	0.056	2.29 (0.98–5.33)	0.278	1.64 (0.67–3.99)	0.072	2.19 (0.93–5.13)
		stage	0.219	1.77 (0.71–4.40)	0.105	2.19 (0.85–5.65)	0.189	1.84 (0.74–4.55)
		age	0.44	1.02 (0.97–1.07)	0.346	1.02 (0.98–1.07)	0.534	1.02 (0.97–1.06)
	UM-HLM	cluster	0.461	1.15 (0.80–1.66)	0.519	1.13 (0.78–1.65)	0.759	1.06 (0.72–1.59)
		stage	<0.0001	2.28 (1.54–3.37)	<0.0001	2.32 (1.57–3.44)	<0.0001	2.31 (1.56–3.41)
		age	1	1.03 (1.01–1.05)	0.002	1.03 (1.01–1.05)	0.002	1.03 (1.01–1.05)
Bladder	AUH	cluster	0.035	2.31 (1.06–5.02)	0.284	1.53 (0.70–3.31)	0.026	2.44 (1.11–5.35)
Ovarium	MNI	cluster	0.016	4.02 (1.30–12.40)	0.002	8.01 (2.12–30.27)	0.219	2.02 (0.66–6.17)
		age	0.094	1.06 (0.99–1.13)	0.035	1.08 (1.01–1.16)	0.069	1.06 (1.00–1.12)

* The direction of the hazard ratio is as follows: cluster, the short-term versus long-term survival group; grade, lower versus higher; age, older versus younger.

a Method A.

b Method B.

c Method C.

**Figure 4:**
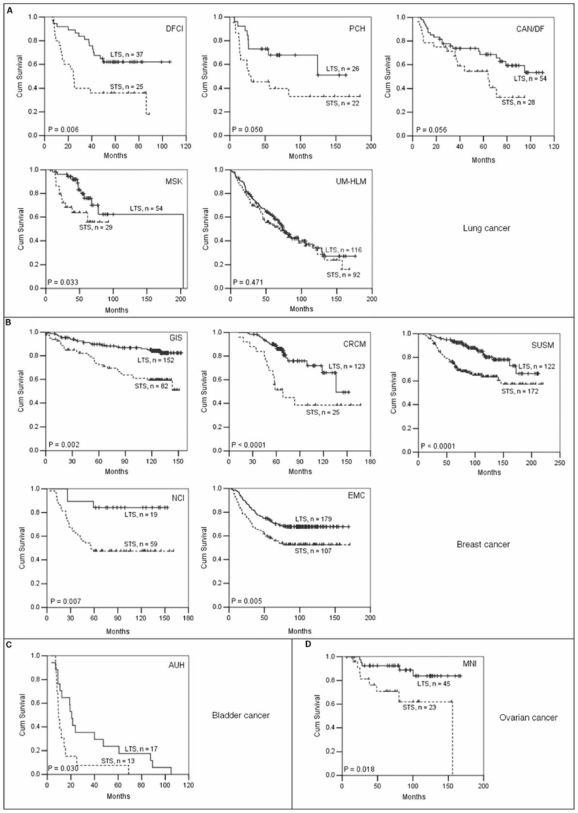
Kaplan-Meier plots of overall survival for other solid tumor types generated by the 23-gene classifier. (A) Five lung cancer cohorts DFCI, PCH, CAN/DF, MSK, and UM-HLM. (B) Five breast cancer cohorts GIS, CRCM, SUSM, NCI and EMC. For NCI and EMC, the overall survival times were unavailable and thus time to distant metastasis for prediction was used instead. (C) One bladder cancer cohort AUH. (D) One ovarian cancer cohort MNI. STS, short-term survival group; LTS, long-term survival group. N, the number of patients within STS or LTS group. *P* values are indicated within plots. *P*< = 0.05 is defined as significance.

We next examined the discriminative power of the 23-gene classifier for survival by Kaplan-Meier analysis in all the 12 cohorts. It significantly classified patients into two different survival groups on 11 of the 12 cohorts except for the cohort UM-HLM-lung cancer (Figure 4A–D). For the four breast cancer cohorts that had stages I, II, and III tumors combined (GIS, CRCM, SUSM and EMC), we also used the 23-gene classifier to classify their patients into three groups by hierarchical clustering, respectively, and found that the three groups in each cohort were also significantly associated with survival (*P* = 0.0001, 0.0001, 0.0001, and 0.01 for GIS, CRCM, SUSM and EMC, respectively).

### Analyses of Additional Classifiers in Advanced Gliomas and Other Tumor Types

To assess the predictive power of previously published prognostic gene signatures for advanced glioma in those cohorts, we tested three main prognostic gene classifiers, 35-gene [4], 44-gene [3], and 47-gene [14] classifiers derived from MDA-HGG, UCLA-HGG, and UCSF-1-GBM, respectively, on the 20 cohorts. To align with the original reports, we divided patients into three (for 35-gene classifier), four (for 44-gene classifier), and two (for 47-gene classifier) groups accordingly. The multivariate Cox regression analysis showed that the associations between the three gene classifiers and survival were much poorer than the 23-gene classifier. The hazard ratios for the 35-gene, 44-gene and 47-gene classifiers were not statistically significant in four, two, and four of the eight advanced glioma cohorts, separately, and eight, nine and 11 of the 12 other tumor cohorts, respectively (Table S15 and S16).

Moreover, we applied the fold-change of at least 1.5 and false discovery rate cutoff of 10% to Method C for identification of candidate prognostic genes for cohorts UCLA, UCSF-1 and MDA, respectively. There was no overlapped prognostic gene identified between any two cohorts. Moreover, we also performed SAM survival analysis as an attempt to identify prognostic genes for the same cohorts. 153 and 137 candidate prognostic genes were identified for cohorts UCSF-1 and UCLA with false discovery rate of 1% and 5%, respectively. For MDA, 59 candidate prognostic genes were found with a false discovery rate of 25%. Based on overlap between any two cohorts and concordant expression across the three cohorts with significant differential expression in at least two cohorts, there was only one gene, “leucine-rich repeats and immunoglobulin-like domains 1” (LRIG1), identified as prognostic gene marker. We also applied univariate Cox regression analysis to Method A to calculate the correlation (S) between expression activity and survival phenotype for prognostic gene identification. As a result, we found 25 prognostic genes, which had 15 genes overlapped with the 23 genes (Table S17). Tests for prediction in all the 20 cohorts showed that both the 25-gene and 15-gene classifiers had less prognostic power than the 23 genes. They failed in three and four of the eight glioma cohorts, separately, and six and nine of the 12 other tumor cohorts, respectively, (Table S18 and S19).

In addition, we performed the gene set approach alone without incorporating the protein interaction network, and identified enriched prognostic gene sets shared in at least two data cohorts with concordant expression (Table S20). Prediction tests showed that the prognostic gene sets with a total of 576 genes had much poorer prognostic power than the 23 genes, failed in 3 of 5 glioma cohorts (Table S21).

### Gene Ontology and Overlap with Other Prognostic Gene Lists

To understand biological functions of the 23 prognostic genes, we performed gene ontology analysis [25] (Table S22). We found that 22 of the 23 genes were significantly associated with either mitotic cell-cycle (EASE score, 1×10^−16^), DNA metabolism (EASE score, 6.27×10^−10^), cell growth and/or maintenance (Ease score, 0.00027), DNA repair (Ease score, 0.0052), or response to stress/immune response (Ease score, 0.019). The only left-out gene is GGH, which is involved in glutamine metabolism. Furthermore, we found that poorer survival was associated with decreased expression of two immune response-related genes, IFNGR1 and “ras-related C3 botulinum toxin substrate 2” (RAC2) and increased expression of 21 genes related to cell cycle (15/23 genes), DNA metabolism (12/23) and repair (4/23), and cell growth/maintenance (16/23), which is consistent with recent reports showing that those biological functions are essential for tumor progression and patient survival [26]–[29].

**Figure 5:**
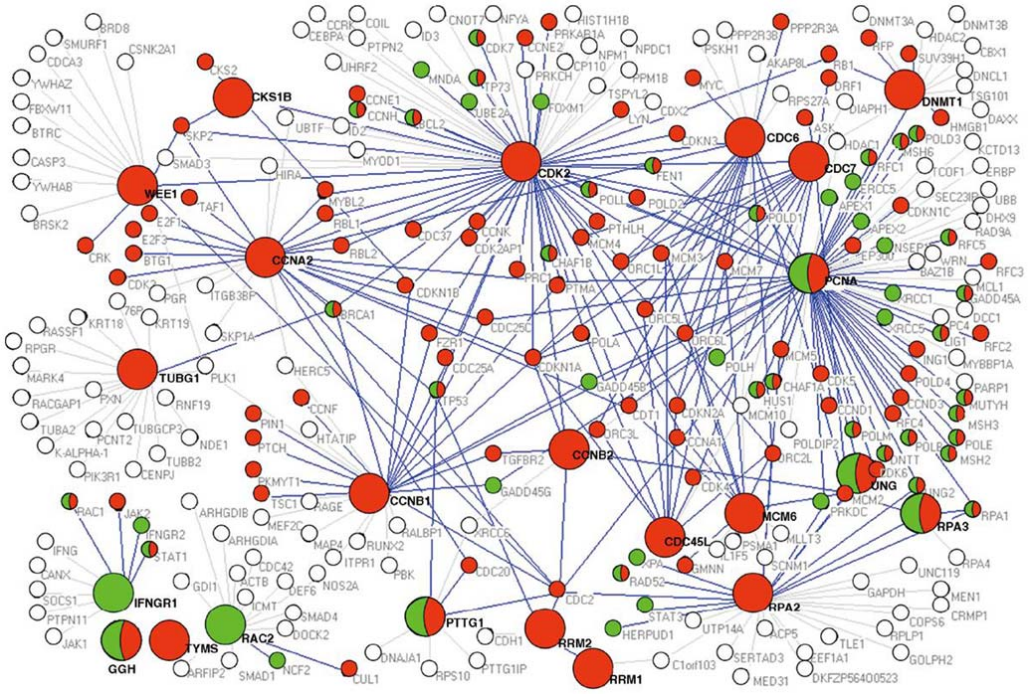
The protein interaction sub-network based on 23 prognostic gene-encoded proteins and their interacting partners. Nodes represent gene-encoded proteins; links represent physical interactions. Nodes in color indicate enriched biological functions of the proteins. Red nodes represent cell proliferation, green nodes represent immune response, and half red and half green nodes represent both cell proliferation and immune response. Proteins in a bigger circle represent the 23 gene-encoded proteins; their interactions with partners with enriched biological functions are highlighted in blue links, whereas grey links represent interactions of the 23 gene-encoded proteins with other partners (white circle).

We then extracted a protein interaction sub-network based on the 23 gene-encoded proteins and their interacting partners from the human protein interaction network. Through GO annotation, we found that the sub-network seeded by 23 genes was related to some important functions, such as cell cycle, regulation of mitosis, regulation of apoptosis, JAK-STAT cascase, MAPKKK cascade, etc (Table S23). Furthermore, we found that most of the interacting proteins were significantly enriched in the biological functions related to cell proliferation and immune response. Those proteins were highlighted in nodes of red (cell proliferation), green (immune response), or half red and half green (both cell proliferation and immune response) (Figure 5).

Finally, we examined the overlap of the 23 prognostic genes with the other prognostic gene lists described above. We found that the 23 genes shared only one gene, CDK2, with 6 genes and 11 genes, and no common gene was found with the additional three glioma prognostic classifiers [3], [4], [14].

## Discussion

The systematic integration of gene sets, the protein interaction network, and microarray gene expression data offers us three main advantages: First, it enables us to sufficiently utilize the gene co-expression information provided by the microarray data, which is believed to be more informative than expression changes of individual genes for target gene identification. Second, identified prognostic genes provide insights into the biology of tumor and potential therapeutic targets. Third, it allows identifying common survival-associated genes independent of tumor types.

The use of the gene sets (Method A) as pre-selected gene sources may introduce some bias and miss an opportunity of finding new survival-associated genes. In the study, we, therefore, directly applied the microarray data to the protein interaction network to first search for significantly enriched sub-networks as proposed by Chuang et al [10], from which we identified prognostic genes (Method B). We found that Method B produced a simpler classifier made up of only 6 genes with 2 in common CDK2 and “replication protein A2” (RPA2) with the 23 genes, and performed slightly less optimally to the 23-gene classifier in the eight glioma cohorts, but very poorly in the 12 other tumor cohorts. This is not surprising as reported by Boutros et al (PNAS 2009) that there exist perhaps hundreds or even thousands of prognostic signatures made up of a small count of genes in large and complex datasets [30]. The power of the 23 genes lies in the prediction for other solid tumors and its potential generality shared among the solid tumors. Moreover, we did not find any new prognostic genes using Method B. Compared with those two gene classifiers, the 11-gene classifier found by Method C (the SAM-based analysis) was obviously incapable of predicting survival in advanced glioma and other tumor types tested, suggesting that the systematic approach with the gene set scale is powerful over gene expression clustering for prognostic gene identification.

When searching for a consensus prognostic gene classifier, some studies have applied a combined (meta-) analysis of several microarray expression data sets and used certain mathematical methods such as Singular value decompositions (SVDs) [31], [32], distance-weighted discrimination (DWD) [33], [34] or analysis of variance (ANOVA) [35] to “correct” systematic biases existed among those data sets to train classifiers [34], [36]. While these methods are certainly a step forward in the right direction, they may bring in some problems as well. Experimental biases present in similar data sets generated in different laboratories using different microarray platforms can be possibly lessened or removed by those methods. However, if data sets contain diverse patient populations, technical and biological effects embedded in the microarray data can not be differentiated. Thus, when applying those methods to ‘correct’ such microarray data, informative biological variability will be removed as well.

In our study, the three training cohorts had very diverse patient populations from the median OS of 14 months (ranged 2–44.54 months) (UCLA), 17 months (0.65–114.85 months) with 17.6% of patients having OS >45 months (UCSF-1) to 17.5 months (0.75–78.25 months) with 18.4% of patients having OS >45 months (MDA). Therefore, we think that it is more reasonable to apply the method (A, B, and C) to each cohort and then reconcile the candidate genes from the three cohorts to reach a final list of prognostic genes based on their overlaps among any two cohorts and concordant expression across the three cohorts. We notice that UCLA has a more biased population with a shorter survival. It may explain why it is difficult to classify the UCLA patients into survival-associated clusters independent of tumor grade and patient age.

Validation of the 23 genes in 20 independent and heterogeneous sample cohorts presented here illustrates the predictive power of the 23-gene classifier independent of tumor grade and patient age across several tumor types. It performs well in either uniform or combined low- or high-grade tumors, indicating that the 23 gene-associated functions (cellular proliferation and immune response) are fundamental and essential for prognosis with both low- and high-grade solid tumors, in other words, cellular proliferation and immune response may be two key prognostic components shared by solid tumors, which is supported by recent findings in breast cancer and lung cancer [26]–[28]. This may explain why the 23-gene classifier performs well in those tumor cohorts tested in this study.

The power of this approach is presently limited by the number of genes in gene pathways and the protein interaction network; however, the prediction performance of the 23-gene signature is impressive, given that only ∼35% of genes are matched in the annotation between our gene sets and the Affymetrix platforms. Future studies will be required to validate the prognostic power of the 23-gene classifier across additional, different tumor types.

We conclude that the systematic approach enables us to identify 23-prognsotic gene classifier that is the first to be valid in 17/20 independent tumor cohorts across several tumor types, suggesting their commonality for solid tumors, especially, for highly proliferating tumors. This approach may also prove useful for other purposes such as for therapeutic response and metastasis.

## Materials and Methods

### Data Collection

We collected microarray gene expression data and clinicopathological information for patients with advanced glioma from three high-grade (HGG) gliomas (grade III and GBM combined) and two GBM data sets publicly available: 74 samples of HGG from UCLA [3], 76 samples of HGG from MDA [4], 50 samples of HGG from CMBC [2], 34 samples of primary GBM from UCSF-1 [14] and 20 samples of GBMs (primary and secondary GBMs combined) from UCSF-2 [15]. We used 118 samples of primary GBM (34 from UCSF-1, 35 from UCLA, and 49 from MDA) as a training set for prognostic gene identification and two independent GBM sample sets from CMBC and UCSF-2 and the three HGG sets from UCLA, MDA, and CMBC for validation. To test the robustness of identified prognostic gene classifiers in different tumor types, we collected 12 completely independent data sets from four different tumor types: five lung cancer sets including 62 stages I-II lung adenocarcinomas from DFCI [21], 48 stage I lung tumors from PCH [22], 82 stages I–II lung adenocarcinomas from CAN/DF [23], 83 stages I–II lung adenocarcinomas from MSK [23], and 208 stages I–II lung adenocarcinomas from UM-HLM [23]; five breast cancer sets including 236 grades I–III breast cancers from GIS [16], 155 grades I–III breast cancers from CRCM [17], 295 grades I–III breast cancers from SUSM [18], 78 stage I breast cancers from NCI [19], and 286 lymph-node-negative breast cancers (mainly stage I) from EMC [20]; one bladder cancer set of 30 advanced bladder cancers from AUH [24]; one ovarian tumor set of 68 stage I ovarian carcinomas from MNI (GEO GSE8842). For the two breast cancer cohorts NCI and EMC, where the overall survival times were unavailable, time to distant metastasis was used instead. For all the cohorts, we used normalized microarray data which are available in public domain (see references). Because several different microarray platforms were used in those cohorts, we ensured that the probes were matched to identical genes. Microarray expression data of prognostic genes identified in this study were further normalized into Z scores prior to clustering. Summary statistics of cohort data sets were presented in Table 1.

### Method A

To integrate gene sets, canonical biological pathways (439 pathways total) were first extracted from the public pathway database MsigDB (http://www.broad.mit.edu/gsea/msigdb/) [37], [38] and then combined with ten human-focused cancer-associated pathways from the Cancer Cell Map (http://cancer.cellmap.org) to form the biggest gene pathways of a total of 449 canonical pathways up to the current date. Further, 2,128 cancer-associated genes [39] were extracted and classified into 403 functional categories using Gene Ontology Consortium analysis (http://www.geneontology.org). After removing overlapped genes between the two gene sources, our 852 gene sets contained 449 gene pathways and 403 functional categories with a total of 5,049 genes. We defined the “significantly enriched gene set” as the gene set that shows statistically significant differences between short-term and long-term survival groups identified by gene set enrichment analysis. Significantly enriched gene sets were then identified by performing gene set enrichment analysis (measuring the degree of differential gene expression in a gene set) between two distinctive survival groups based on microarray expression data using SAM-GS software [8].

Significantly enriched gene sets were next mapped to the human protein-protein interaction network that has 9,213 genes and 37,107 interactions [40] to obtain an enriched gene sub-network. Survival-associated genes were identified by sequentially scoring the genes in the enriched sub-network. Specifically, given a gene *G*, let *E* represent its vector of expression scores over tumor samples, and let *T* represent the corresponding vector of survival phenotype. To derive *E*, the expression values of gene *G* and its nearest neighbor genes in the enriched sub-network were normalized over all samples (mean = 0; s.d. = 1). The normalized expression values of gene *G* and its neighbor genes were averaged into a combined score, designated as *E*. The correlation between *E* and *T*, denoted as *S*, was calculated by Pearson correlation analysis. The null distribution of *S* was estimated by permuting the survival phenotype 100,000 times. The final score of gene *G* was indexed on this null distribution. Because the Bonferroni correction used to adjust for multiple comparisons is often too concervative when applied to microarray data [41], the less stringent Benjamini and Hochberg false discovery rate [42] was performed for multiple comparison correction. A significant survival-associated gene was identified when the corrected *P* value of the correlation was less than 0.05.

### Method B

Method B was divided into two steps. The first was to identify significantly enriched sub-networks using microarray gene expression data and the protein interaction network as described by Chuang et al [10]. Briefly, expression values of genes from microarray expression analysis were directly overlaid on their corresponding proteins in the protein interaction network (7,683 genes from the training sets UCLA and MDA and 6046 genes from the training set UCSF-1) to search for enriched sub-networks by calculating the discriminative score (student's t test) of the relationship between expression activity of each sub-network and survival phenotype. Sub-network started from a seed protein and iteratively expanded by adding a protein from the neighbors of the seed protein until no addition increased the discriminative score. The significance of the discriminative score was estimated by permuting survival phenotype 100,000 times. The final score of sub-network was indexed on this null distribution. A significantly enriched (differentially expressed) sub-network was identified when the *P* value of relationship was less than 0.05.

Secondly, to identify survival-associated genes from significantly enriched sub-networks, an expression value of each gene in the enriched sub-networks was z-transformed over all samples and the association between its z-transformed value and survival was then assessed by univariate Cox regression analysis. The less stringent Benjamini and Hochberg false discovery rate [42] was performed for multiple comparison correction. A prognostic gene was identified when the corrected *P* value of the association was less than 0.05.

### Method C

Differentially expressed genes between two distinctive survival groups were directly identified by two-class analysis (students' t test) of microarray gene expression data using the SAM software. SAM output data were presented along with the false discovery rate. The same number of top discriminative genes was selected as the number of genes identified by method A while, where possible, the lowest false discovery rate was adopted.

### Survival Prediction

Multivariate Cox proportional-hazards regression analysis with stepwise selection was used to evaluate independent prognostic factors associated with survival, and gene expression cluster defined by gene classifier (or subsets of gene classifier), tumor grade or stage, and age were used as covariates. For each covariate, a hazard ratio and an associated *P* value were examined. The Kaplan-Meier method was used to estimate overall survival distribution and metastasis-free survival distribution (for two breast cancer cohorts). Differences in survival between distinctive survival groups were analyzed with the log-rank test. A *P* value of less than 0.05 was considered to indicate statistical significance, and all tests were two-tailed. Statistical analyses were carried out using SPSS software version 13.

As for testing three glioma prognostic gene classifiers reported previously [3], [4], [14], the 35-gene, 44-gene and 47-gene classifiers divided patients into three, four and two prognostic groups, respectively, in our cohorts to be consistent with their original studies.

### Gene Ontology Analysis

Gene functions and their biological significance (EASE score) were assessed by using the EASE annotation tool (EASE software version 2.0) [25]. Fisher's exact test combined with Bonferroni correction was used to calculate the significance.

## Supporting Information

Figure S1 Kaplan-Meier plots of overall survival for primary GBMs using the 6-genes.(A) MDA; (B) UCLA; (C) UCSF-1. P values are indicated within plots. P< = 0.05 is defined as significance. STS is short-term survival group; LTS is long-term survival group; n is the number of patients within STS or LTS group.
(0.06 MB PDF)

Figure S2 Kaplan-Meier plots of overall survival for glioma using the 6-genes. (A) Two GBM cohorts UCSF-2 and CMBC. (B) Three HGG cohorts MDA, UCLA, and CMBC. P values are indicated within plots. P< = 0.05 is defined as significance. STS is short-term survival group; LTS is long-term survival group; n is the number of patients within STS or LTS group.
(0.09 MB PDF)

Figure S3 Kaplan-Meier plots of overall survival for glioma using the 6-genes. (A) Five lung cancer cohorts DFCI, PCH, CAN/DF, MSK, and UM-HLM. (B) Five breast cancer cohorts GIS, CRCM, SUSM, NCI and EMC. (C) One bladder cancer cohort AUH. (D) One ovarian cancer cohort MNI. P values are indicated within plots. P< = 0.05 is defined as significance. STS is short-term survival group; LTS is long-term survival group; n is the number of patients within STS or LTS group.
(0.23 MB PDF)

Figure S4 Kaplan-Meier plots of overall survival for primary GBMs using the 11-genes. (A) MDA; (B) UCLA; (C) UCSF-1. P values are indicated within plots. P< = 0.05 is defined as significance. STS is short-term survival group; LTS is long-term survival group; n is the number of patients within STS or LTS group.
(0.06 MB PDF)

Figure S5 Kaplan-Meier plots of overall survival for glioma using the 11-genes. (A) Two GBM cohorts UCSF-2 and CMBC. (B) Three HGG cohorts MDA, UCLA, and CMBC. P values are indicated within plots. P< = 0.05 is defined as significance. STS is short-term survival group; LTS is long-term survival group; n is the number of patients within STS or LTS group.
(0.09 MB PDF)

Figure S6 Kaplan-Meier plots of overall survival for glioma using the 11-genes. (A) Five lung cancer cohorts DFCI, PCH, CAN/DF, MSK, and UM-HLM. (B) Five breast cancer cohorts GIS, CRCM, SUSM, NCI and EMC. (C) One bladder cancer cohort AUH. (D) One ovarian cancer cohort MNI. P values are indicated within plots. P< = 0.05 is defined as significance. STS is short-term survival group; LTS is long-term survival group; n is the number of patients within STS or LTS group.
(0.23 MB PDF)

Table S1 124 significantly enriched gene sets identified from primary GBM cohorts of MDA
(0.11 MB XLS)

Table S2 114 significantly enriched gene sets identified from primary GBM cohorts of UCSF-1
(0.10 MB XLS)

Table S3 78 significantly enriched gene sets identified from primary GBM cohorts of UCLA
(0.07 MB XLS)

Table S4 List of candidate survival-associated genes developed by method A from primary GBM data in UCLA.
(0.01 MB PDF)

Table S5 List of candidate survival-associated genes developed by method A from primary GBM data in UCSF-1.
(0.03 MB PDF)

Table S6 List of candidate survival-associated genes developed by method A from primary GBM data in MDA.
(0.03 MB PDF)

Table S7 List of candidate survival-associated genes developed by method B from primary GBM data in UCLA.
(0.03 MB PDF)

Table S8 List of candidate survival-associated genes developed by method B from primary GBM data in UCSF-1.
(0.03 MB PDF)

Table S9 List of candidate survival-associated genes developed by method B from primary GBM data in MDA.
(0.03 MB PDF)

Table S10 List of prognostic genes developed by method B from primary GBM data in UCLA, UCSF-1, and MDA.
(0.01 MB PDF)

Table S11 List of candidate survival-associated genes developed by method C from primary GBM data in UCLA.
(0.01 MB PDF)

Table S12 List of candidate survival-associated genes developed by method C from primary GBM data in UCSF-1.
(0.03 MB PDF)

Table S13 List of candidate survival-associated genes developed by method C from primary GBM data in MDA.
(0.03 MB PDF)

Table S14 List of prognostic genes developed by method C from primary GBM data in UCLA, UCSF-1, and MDA.
(0.01 MB PDF)

Table S15 Multivariate cox regression analysis of previously reported prognostic genes for glioma in training and validation cohorts of glioma.
(0.01 MB PDF)

Table S16 Multivariate cox regression analysis of previously reported prognostic genes for glioma in training and validation cohorts of other tumors.
(0.01 MB PDF)

Table S17 25 prognostic genes developed by method A (Cox regression) from primary GBM data in UCLA, UCSF-1, and MDA. 15 prognostic genes are overlapped genes between 25 prognostic genes and 23 prognostic genes (see Table 2 in the manuscript).
(0.03 MB PDF)

Table S18 Multivariate cox regression analysis of 25 genes and 15 genes in training and validation cohorts of glioma.
(0.01 MB PDF)

Table S19 Multivariate cox regression analysis of 25 genes and 15 genes in training and validation cohorts of other tumors.
(0.01 MB PDF)

Table S20 Prognostic gene sets
(0.04 MB XLS)

Table S21 Multivariate cox regression analysis of prognostic gene sets in training and validation cohorts of glioma.
(0.01 MB PDF)

Table S22 Biological functions of 23 prognostic genes.
(0.02 MB XLS)

Table S23 Biological functions of the protein interaction sub-network seeded by the 23 prognostic genes
(0.03 MB XLS)
