# Supplementary figures and images for: A Systems Biology-Based Gene Expression Classifier of Glioblastoma Predicts Survival with Solid Tumors
Source: PLoS One. 2009 Jul 17;4(7):e6274. doi: 10.1371/journal.pone.0006274 (PMC2707631; doi:10.1371/journal.pone.0006274)

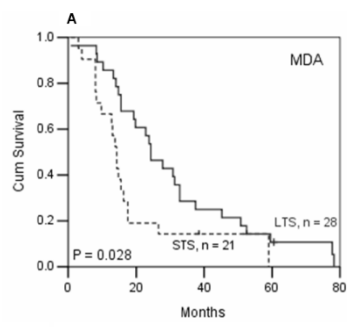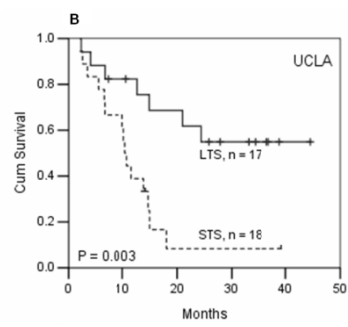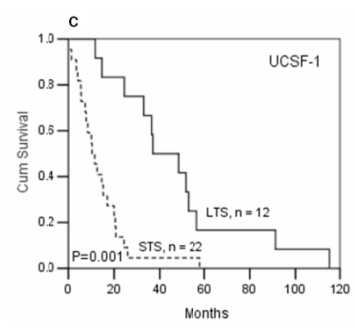

Supplement: Figure S1 — Kaplan-Meier plots of overall survival for primary GBMs using the 6-genes.(A) MDA; (B) UCLA; (C) UCSF-1. P values are indicated within plots. P< = 0.05 is defined as significance. STS is short-term survival group; LTS is long-term survival group; n is the number of patients within STS or LTS group. (0.06 MB PDF) [file pone.0006274.s001.pdf]

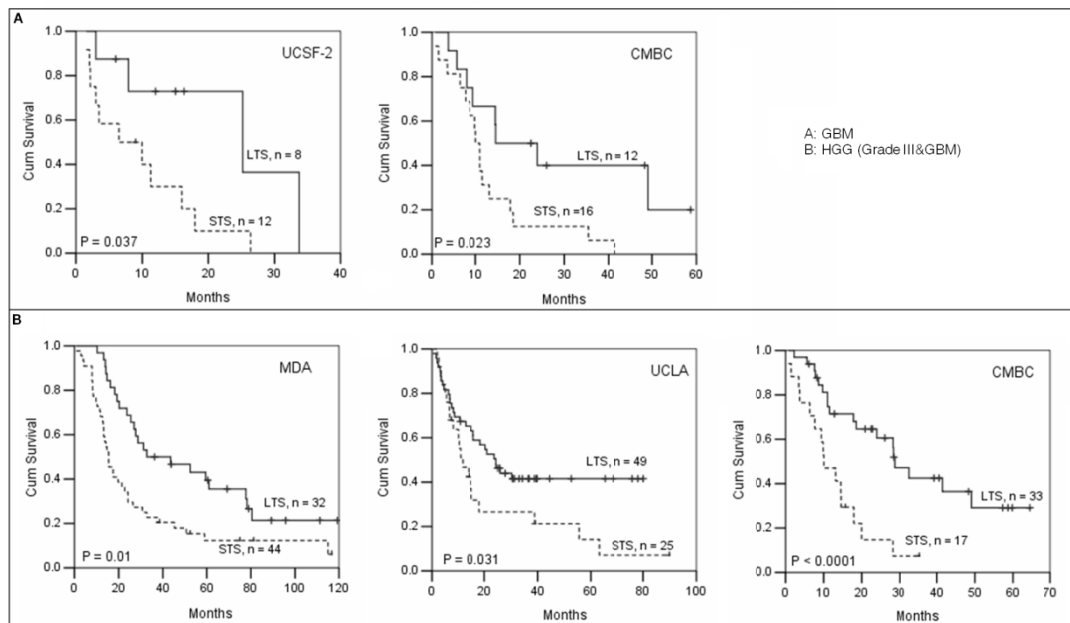

Supplement: Figure S2 — Kaplan-Meier plots of overall survival for glioma using the 6-genes. (A) Two GBM cohorts UCSF-2 and CMBC. (B) Three HGG cohorts MDA, UCLA, and CMBC. P values are indicated within plots. P< = 0.05 is defined as significance. STS is short-term survival group; LTS is long-term survival group; n is the number of patients within STS or LTS group. (0.09 MB PDF) [file pone.0006274.s002.pdf]

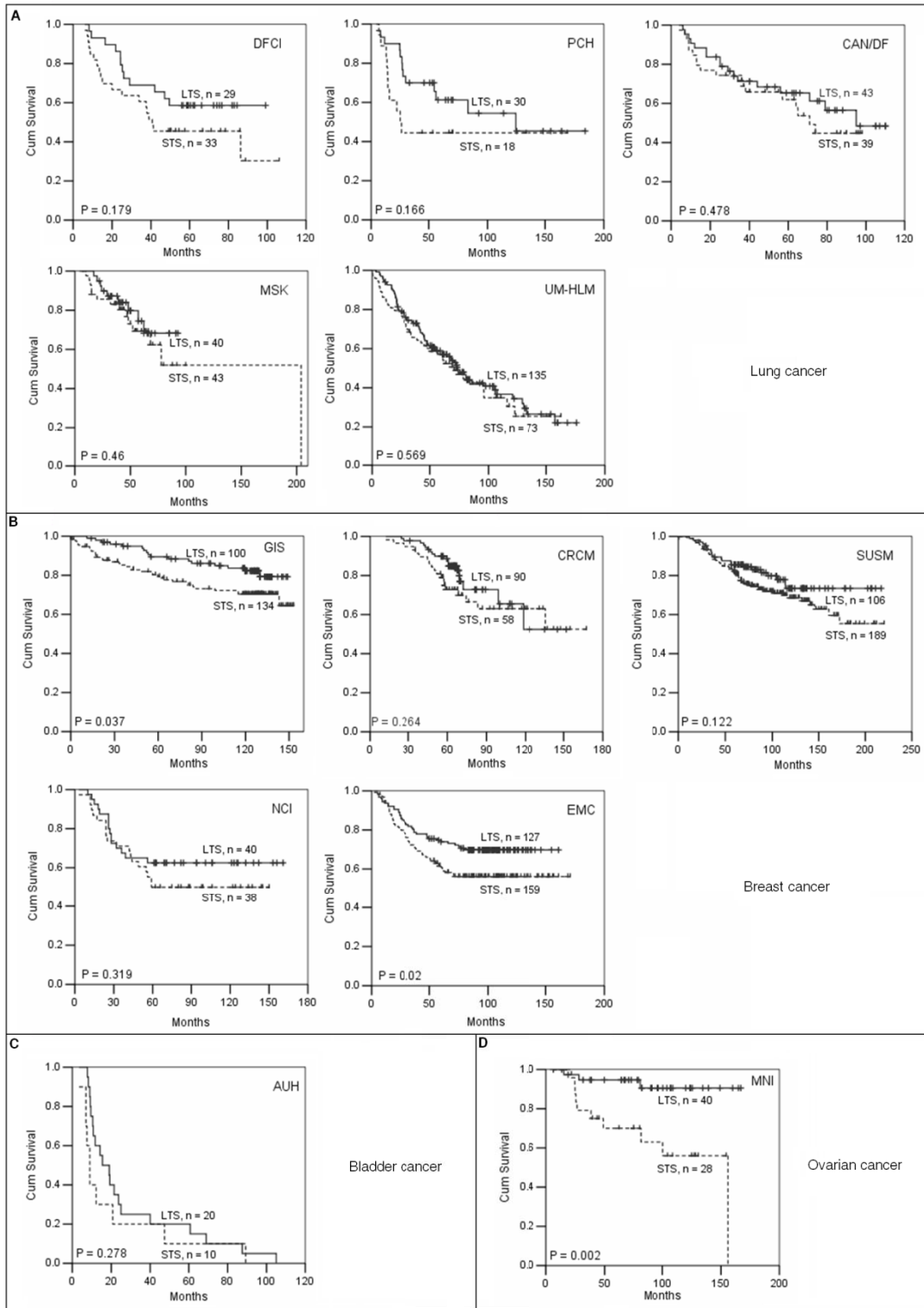

Supplement: Figure S3 — Kaplan-Meier plots of overall survival for glioma using the 6-genes. (A) Five lung cancer cohorts DFCI, PCH, CAN/DF, MSK, and UM-HLM. (B) Five breast cancer cohorts GIS, CRCM, SUSM, NCI and EMC. (C) One bladder cancer cohort AUH. (D) One ovarian cancer cohort MNI. P values are indicated within plots. P< = 0.05 is defined as significance. STS is short-term survival group; LTS is long-term survival group; n is the number of patients within STS or LTS group. (0.23 MB PDF) [file pone.0006274.s003.pdf]

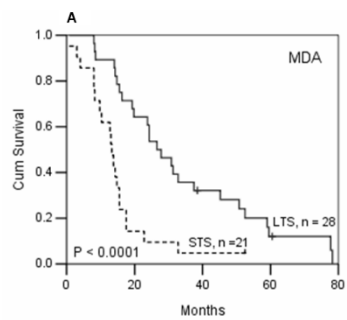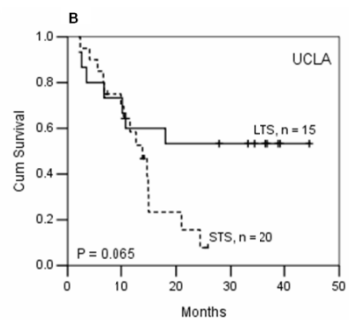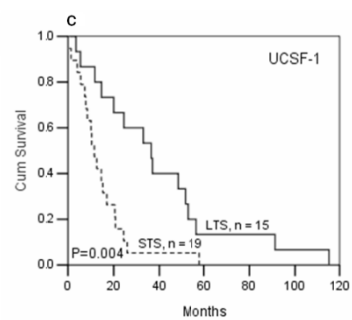

Supplement: Figure S4 — Kaplan-Meier plots of overall survival for primary GBMs using the 11-genes. (A) MDA; (B) UCLA; (C) UCSF-1. P values are indicated within plots. P< = 0.05 is defined as significance. STS is short-term survival group; LTS is long-term survival group; n is the number of patients within STS or LTS group. (0.06 MB PDF) [file pone.0006274.s004.pdf]

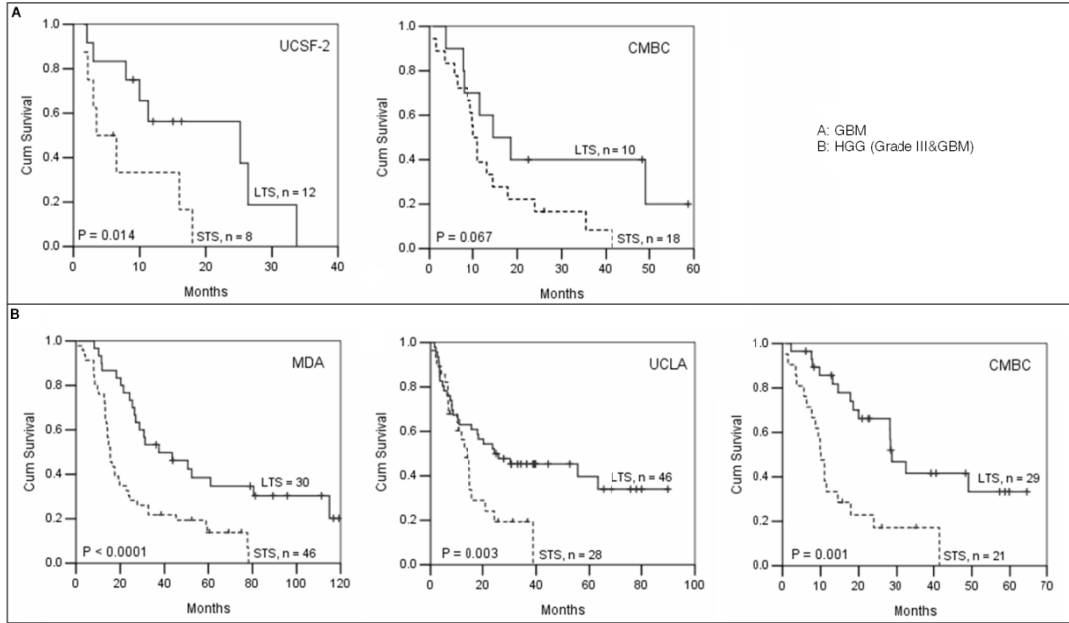

Supplement: Figure S5 — Kaplan-Meier plots of overall survival for glioma using the 11-genes. (A) Two GBM cohorts UCSF-2 and CMBC. (B) Three HGG cohorts MDA, UCLA, and CMBC. P values are indicated within plots. P< = 0.05 is defined as significance. STS is short-term survival group; LTS is long-term survival group; n is the number of patients within STS or LTS group. (0.09 MB PDF) [file pone.0006274.s005.pdf]

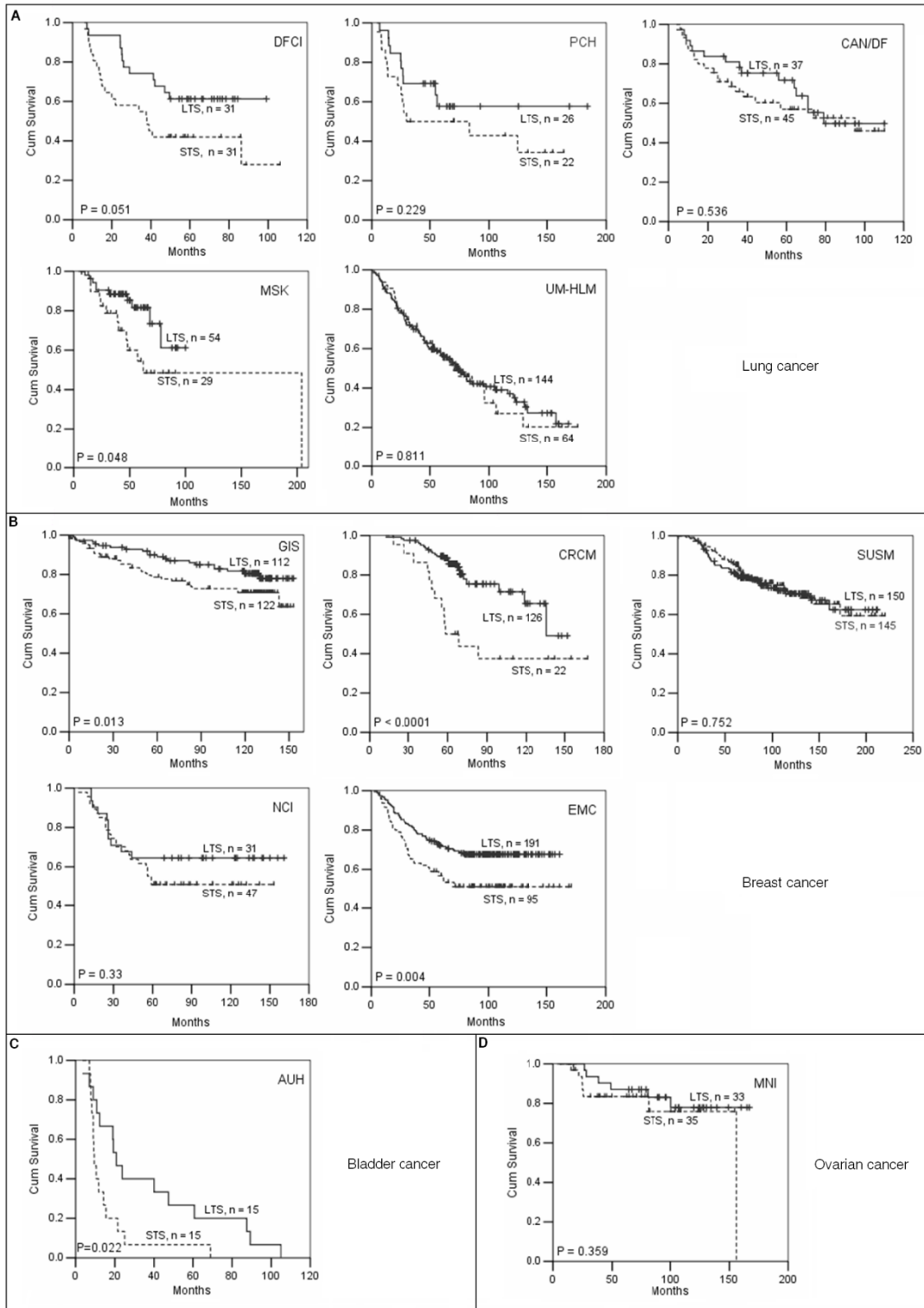

Supplement: Figure S6 — Kaplan-Meier plots of overall survival for glioma using the 11-genes. (A) Five lung cancer cohorts DFCI, PCH, CAN/DF, MSK, and UM-HLM. (B) Five breast cancer cohorts GIS, CRCM, SUSM, NCI and EMC. (C) One bladder cancer cohort AUH. (D) One ovarian cancer cohort MNI. P values are indicated within plots. P< = 0.05 is defined as significance. STS is short-term survival group; LTS is long-term survival group; n is the number of patients within STS or LTS group. (0.23 MB PDF) [file pone.0006274.s006.pdf]
