# Supplementary material for: A Systems Biology-Based Gene Expression Classifier of Glioblastoma Predicts Survival with Solid Tumors
Source: PLoS One. 2009 Jul 17;4(7):e6274. doi: 10.1371/journal.pone.0006274 (PMC2707631; doi:10.1371/journal.pone.0006274)
Supplement: Table S4 — List of candidate survival-associated genes developed by method A from primary GBM data in UCLA. (0.01 MB PDF) [file pone.0006274.s010.pdf]

Table S4 . List of candidate survival-associated genes developed by method A from primary GBM data in UCLA.

Gene Symbol	Score	Gene Symbol	Score	Gene Symbol	Score	Gene Symbol	Score
NOTCH2	0.0001	ETS1	0.0089	PTPRU	0.0213	JUN	0.0381
PLCB1	0.0001	SKI	0.0091	ZAP70	0.0216	RRAS2	0.0381
PARD3	0.0002	CDC14B	0.0092	CCNB1	0.0217	FZD7	0.0384
MAPK4	0.0003	NFYC	0.0095	TCF1	0.0219	EMR1	0.0391
JUND	0.0005	ASCL1	0.0095	IL13RA2	0.0219	RPS5	0.0391
ADK	0.0006	BMPR1A	0.0098	PTPN6	0.0223	RBPSUH	0.0392
CRKL	0.0006	GAB1	0.01	TUBG1	0.0225	EIF4B	0.0402
CALCRL	0.001	CDC42	0.01	PHF11	0.0227	PLK1	0.0403
ARRB2	0.001	DAPK1	0.0103	SOS1	0.0227	JAG1	0.0405
GGH	0.0013	CAPZA1	0.0105	FZD8	0.0234	NCOR1	0.0405
IRS1	0.0016	HES1	0.0108	GCH1	0.0234	RPL28	0.0408
NOTCH1	0.0017	CCNE1	0.0109	PDGFRA	0.0236	FHL1	0.0411
FKBP1A	0.0017	RPS6KB2	0.0111	CCNE2	0.0238	GNA12	0.0413
HDAC5	0.0018	EIF3S10	0.0113	PRKD1	0.024	PLCG2	0.0415
RAF1	0.003	TMSB10	0.0115	RAC3	0.0243	PTGS1	0.0415
RPS6KA4	0.0034	CHP	0.0115	GNB1	0.0243	FADD	0.0416
LDHA	0.0035	KRT19	0.0121	GLP2R	0.0244	ACVR1	0.0416
SH3BGRL	0.0036	CTCF	0.0126	CNTN1	0.0251	RELA	0.0417
PTPN11	0.0037	PEA15	0.0126	PPP2CA	0.0252	GUCY2D	0.0419
MAPK10	0.0046	GAB2	0.0129	DNAH9	0.026	STK11	0.0423
GATA1	0.0046	PITX2	0.0131	GLP1R	0.0261	INPP5D	0.0423
MAP4K5	0.005	GATA4	0.0134	PLA2G2A	0.0265	CEBPB	0.0424
COPS5	0.0051	TCF8	0.0142	PAK2	0.0279	PAFAH1B1	0.0432
PPP3CA	0.0054	PCAF	0.0143	JAG2	0.0294	MAPK8	0.0435
MT1H	0.0055	ESR1	0.0149	RAC2	0.0307	PLD2	0.044
MAP3K2	0.006	PTHR1	0.0151	TRIP10	0.031	VAV1	0.0451
SNX4	0.006	GRB2	0.0152	ORC6L	0.0325	TIAM1	0.0457
HCK	0.0066	PRKCD	0.0153	PFDN4	0.0327	CDC45L	0.0459
PIK3CB	0.0068	PRKCG	0.0153	NOTCH3	0.0328	CRK	0.0459
RPS6KA3	0.0069	SOCS3	0.0154	ITPR3	0.033	GADD45G	0.0462
STK17B	0.0071	PRPS1	0.0156	BAX	0.0332	CALM3	0.0463
MT1G	0.0071	MAML1	0.0175	FOS	0.0332	ZFHX1B	0.0464
PRKCE	0.0072	FRAP1	0.0175	RAB26	0.0337	NUDT4	0.0465
TNFRSF1A	0.0078	IL3RA	0.0176	TCF12	0.0341	MCM6	0.0467
TYMS	0.008	BDH	0.0176	TGFBRAP1	0.035	PTK2	0.0468
ITPR1	0.008	PRKAR2A	0.0178	PRKAR1A	0.0351	ABL1	0.0479
TSC2	0.0081	RAB9A	0.0183	RBX1	0.0362	IFNGR1	0.0482
DHFR	0.0084	STAT2	0.0187	MAP2K2	0.0363	ZNF318	0.0483
S100B	0.0084	FOXO3A	0.0191	GDI2	0.0367	NCK2	0.0489
HDAC4	0.0088	TYK2	0.0196	TEC	0.037	IFNAR2	0.0495
CAMK2G	0.0474	SYK	0.0198	S100A1	0.0373	PTTG3	0.05
