# Supplementary material for: A Systems Biology-Based Gene Expression Classifier of Glioblastoma Predicts Survival with Solid Tumors
Source: PLoS One. 2009 Jul 17;4(7):e6274. doi: 10.1371/journal.pone.0006274 (PMC2707631; doi:10.1371/journal.pone.0006274)
Supplement: Table S5 — List of candidate survival-associated genes developed by method A from primary GBM data in UCSF-1. (0.03 MB PDF) [file pone.0006274.s011.pdf]

Table S5. List of candidate survival-associated genes developed by method A from primary GBM data in UCSF-1.

Gene Symbol	Score	Gene Symbol	Score	Gene Symbol	Score	Gene Symbol	Score
NEFL	0	CKS1B	0.006	MAT1A	0.014	CASP6	0.0234
MAP2K4	0.0002	ATP6V1C1	0.0062	TRIP10	0.0141	GABRA2	0.024
RGS11	0.0003	CYCS	0.0062	HMGB2	0.0143	RRM2	0.0242
AP1S2	0.0005	ATP6V0C	0.0063	ATP6V1H	0.0143	CYP51A1	0.0242
NF1	0.0005	DUSP4	0.0063	GPI	0.0144	PSMD8	0.0243
HRAS	0.0006	DNMT1	0.007	ETF1	0.0147	UNG	0.0246
PRKAR2B	0.0007	BCKDK	0.0073	ID4	0.015	ARF4	0.0246
CFLAR	0.0009	MAPK8IP2	0.0073	EIF3S6	0.0151	CYC1	0.0253
CTSS	0.001	RRAS	0.0073	AKAP4	0.0151	DHFR	0.0254
CACNB1	0.0014	CDKN1B	0.0076	TRAF1	0.0155	DTYMK	0.0261
MAD2L1	0.0015	CASP2	0.0076	RPA3	0.0158	PDE6C	0.0262
MAPK8IP3	0.0017	CAMK2A	0.0077	SPTAN1	0.0162	KRT14	0.0274
POLD2	0.0018	SKP2	0.0079	PDK1	0.0163	ALDH2	0.0278
EIF2B5	0.0019	CCT2	0.008	ID3	0.0165	FBP2	0.0282
MAPK8IP1	0.0021	FLT1	0.0081	TK1	0.0165	AKAP6	0.0284
GRIN1	0.0023	PTTG1	0.0083	MXI1	0.0176	RPA2	0.0288
GGH	0.0025	MAFK	0.0087	CDC6	0.0176	ALDH1A1	0.0289
HDAC2	0.0026	BIRC3	0.0088	TYMS	0.0178	CDKN2C	0.0291
PPARG	0.0027	CYP1A1	0.009	PHKG2	0.0179	CUL4A	0.0308
PRKAR2A	0.0028	EIF2S1	0.0091	CDC23	0.0179	CDC2	0.0311
ADH1B	0.0028	DGAT1	0.0094	ID2	0.018	ARHGAP4	0.0313
CDC20	0.0029	CDKN1A	0.0094	HSPA1L	0.0183	MARS	0.0315
PICALM	0.003	RASA1	0.0095	TUBB4	0.0185	PAX5	0.0321
MAP2K7	0.0031	NR2F1	0.0098	PPP3CA	0.0188	FAT	0.0322
GRIN2D	0.0031	MAPK11	0.0099	ARHGAP6	0.0192	EEF1A1	0.0324
RAG1	0.0036	EIF2S2	0.0101	UMPS	0.0192	PIN1	0.0327
CABIN1	0.0038	PSEN2	0.0102	SKIP	0.0199	GPRC5B	0.0331
PRKCH	0.0038	PGK1	0.0103	CDH16	0.02	SNAP25	0.0334
POLA	0.004	NRAS	0.0106	PPARBP	0.0204	ATF5	0.0336
BMP4	0.004	NSF	0.0111	CDC16	0.0205	AMPH	0.0337
CDH18	0.0041	VAMP2	0.0112	MOG	0.0205	NDP	0.0343
ENDOG	0.0046	RRM1	0.0114	CACNA1A	0.021	CCNA2	0.0346
ARNT	0.0048	PDGFB	0.0116	NDST2	0.0212	ADAM2	0.0348
CASP10	0.0048	ELA2A	0.0119	CRADD	0.0215	PDE4D	0.035
CCNB2	0.0049	DES	0.0124	CDH1	0.0219	BCL2L1	0.0359
GABARAP	0.005	ATP6V1G2	0.0124	RB1	0.022	HDAC4	0.036
CDK2	0.005	RPS6KA3	0.0125	GNGT1	0.0226	PLA2G6	0.036
GLA	0.0054	SLC9A3R1	0.0131	PCNA	0.0227	FDX1	0.0361
PTMA	0.0057	GRM3	0.0132	PDE6H	0.0228	CAMK2G	0.0461
PDCD8	0.0059	HDAC6	0.0132	CDC7	0.0229	MCM6	0.0484
AHCY	0.006	AKAP9	0.0132	ALPI	0.023	MUSK	0.0493
ADD1	0.037	NTSR1	0.0389	TGFB1	0.0426	EEF1A2	0.0492
CDKN2A	0.0373	MCL1	0.0389	HES1	0.043	GNAL	0.0468
BUB1B	0.0375	AKAP5	0.0391	PTTG2	0.043	CREB1	0.0426
EIF2B2	0.0377	MMP7	0.0394	CHGB	0.0436	POLE	0.0389
ASK	0.0378	MAP1B	0.0395	PPP1R1A	0.0436	TERF2IP	0.0468
PTN	0.0379	TBXAS1	0.0399	EIF3S2	0.0451	HK2	0.0426
CDC45L	0.0379	RBL1	0.0413	BID	0.0453	PSMB8	0.0478
RPA1	0.038	WEE1	0.0425	BAD	0.0458	THRA	0.05
IL11	0.0381	BIN1	0.0426				
