# Supplementary material for: A Systems Biology-Based Gene Expression Classifier of Glioblastoma Predicts Survival with Solid Tumors
Source: PLoS One. 2009 Jul 17;4(7):e6274. doi: 10.1371/journal.pone.0006274 (PMC2707631; doi:10.1371/journal.pone.0006274)
Supplement: Table S6 — List of candidate survival-associated genes developed by method A from primary GBM data in MDA. (0.03 MB PDF) [file pone.0006274.s012.pdf]

Table S6. List of candidate survival-associated genes developed by method A from primary GBM data in MDA.

Gene Symbol	Score	Gene Symbol	Score	Gene Symbol	Score	Gene Symbol	Score
POLL	0.0001	FCER1G	0.0072	ESPL1	0.0196	TLR7	0.0369
PTHLH	0.0004	CCNA1	0.0073	RBL2	0.0196	CCL4	0.0371
KRT15	0.0007	RRM1	0.0075	TP53	0.0196	PECAM1	0.0371
BTG3	0.0007	POLE	0.0077	HPRT1	0.0201	ADD1	0.0378
POLD2	0.0007	MYBL2	0.0077	RRM2	0.0204	FOLR1	0.0378
WEE1	0.0008	ORC4L	0.0078	CDH1	0.0207	CR2	0.038
CHEK2	0.001	SKP2	0.0084	DLG7	0.0209	TRIP10	0.0381
BRIP1	0.0016	YY1	0.0085	CCNH	0.021	CSK	0.0381
CCR1	0.0017	DNMT3B	0.0088	CDC2	0.0211	EYA4	0.0385
RAD51C	0.0019	RFC1	0.0088	CCL2	0.0214	RPA4	0.0393
CR1	0.0019	HIST1H1B	0.0089	POLD3	0.0214	NXT1	0.0403
CCNA2	0.0019	ORC5L	0.009	DNM1	0.0218	MCM7	0.0403
CKS1B	0.002	GNB5	0.009	BTK	0.0219	DUSP3	0.0405
GMNN	0.002	CD79A	0.0091	TRRAP	0.022	E2F6	0.0406
CCNB2	0.002	MITF	0.0094	PTPRC	0.0227	HDAC1	0.0412
RAD51L3	0.0021	MCM5	0.0094	SMC1L1	0.0229	PMS1	0.0413
SSTR2	0.0021	MCM3	0.0096	SHMT1	0.0231	CDC25C	0.0414
CDT1	0.0021	CDK7	0.0097	ITM2B	0.0232	KIF2C	0.0415
MCM4	0.0022	CTPS	0.0099	CRADD	0.0233	ARHGDIB	0.0417
CDC5L	0.0023	HDAC2	0.01	DCTD	0.0236	CBL	0.0422
CDK3	0.0024	CRYAB	0.01	FAT	0.0237	WTAP	0.0431
IFNA1	0.0024	DNMT1	0.01	CDKN2A	0.0238	LY96	0.0432
CDC7	0.0024	AK2	0.01	CDK4	0.024	CASP2	0.0437
FLJ14001	0.0025	ORC6L	0.0101	IFNGR1	0.0241	RAG1	0.0442
ASK	0.0026	PCNA	0.0101	UBE1	0.0242	DNAJA1	0.0444
UNG	0.0027	TYK2	0.0106	PTPRU	0.0247	IRF1	0.0446
TFIP11	0.0028	PLCG2	0.0106	MYD88	0.0247	ANXA8	0.0449
BRCA2	0.003	SERPINA3	0.0107	TNFRSF8	0.0248	RXRA	0.0455
RUVBL2	0.0031	CD19	0.0109	KRT14	0.0249	ITPR2	0.0457
ORC1L	0.0031	PTAFR	0.0109	CD72	0.025	TRAF6	0.0457
TFDP2	0.0032	PSEN2	0.011	FH	0.0252	CXCR4	0.0461
RPA3	0.0032	HUS1	0.0111	ORC3L	0.0253	ITGB2	0.0461
CDC6	0.0032	RBL1	0.0111	GPR30	0.0254	NFKBIL1	0.0469
CSF2RB	0.0033	PAWR	0.0113	GMPS	0.0256	C3	0.0469
CDC45L	0.0033	EEF1A1	0.0115	ICAM1	0.0256	S100A7	0.0471
TIMP3	0.0034	POLA	0.012	GJA1	0.0266	TOP2B	0.0487
MCM10	0.0037	TNFRSF1A	0.012	APEX1	0.0269	CAMK2G	0.049
XRCC3	0.0038	RFC2	0.0122	BRCA1	0.0276	FOXO3A	0.0494
CIRBP	0.0039	TACSTD2	0.0122	POLR2D	0.0278	POLQ	0.0498
POLR2B	0.0039	E2F1	0.0124	DHFR	0.0279	PTPN6	0.0195
MCM6	0.0039	VCL	0.0128	RAD51L1	0.028	CCNB1	0.0189
POLR2K	0.004	KLK7	0.0128	CD14	0.0281	TYMS	0.0189
IFITM1	0.0041	KNTC1	0.0129	OSM	0.0282	RAD17	0.019
RAD51	0.0041	ATM	0.013	PTTG1	0.0284	ISGF3G	0.0191
FEN1	0.0042	MSH2	0.0132	PRIM1	0.0293	EPN1	0.0192
RB1	0.0042	RFC4	0.0135	TEC	0.03	PTPN11	0.0194
MCM2	0.0042	PRTN3	0.0135	H1F0	0.0303	PMS2	0.0182
ORC2L	0.0043	CCNE2	0.0136	SHMT2	0.0306	RAC1	0.0183
CDKN3	0.0044	CDC25A	0.0142	BIRC5	0.031	CCR3	0.0184
NFKB2	0.0045	DAPP1	0.0142	CCT2	0.0313	BRD2	0.0186
POLA2	0.0045	CDK6	0.0149	BUB1	0.0313	PIN1	0.007
WRN	0.0045	FANCE	0.015	LRP2	0.032	EXO1	0.0066
WT1	0.0045	DNMT3A	0.0152	RPS6KA1	0.0325	FCGR2B	0.0179
CDK2	0.0046	DUSP12	0.0154	RAF1	0.0327	POLD1	0.0064
CYP3A5	0.0046	SYK	0.0154	TNFRSF1B	0.0334	MSH6	0.0177
ITPKB	0.0049	CREBBP	0.0156	PTK2B	0.0335	RPA2	0.0062
RAC2	0.0049	SSTR1	0.0158	IFNAR2	0.0336	CDKN1A	0.0177
RFC5	0.0051	CSF1	0.0161	GJB1	0.034	TOB2	0.0176
RPA1	0.0052	HLA-DRB1	0.0168	ITPKA	0.0344	RUVBL1	0.0061
BLM	0.0052	HLA-DRA	0.0168	FANCF	0.0348	CD79B	0.0176
DAXX	0.0053	LATS1	0.0172	TLK1	0.035	ATR	0.0365
CCR5	0.0055	DNAJC7	0.0173	GNAQ	0.0353	PTMA	0.006
MUTYH	0.0055	PLK1	0.0173	FANCG	0.0357	FCER1A	0.0364
TUBG1	0.0055	TFDP1	0.0175	PML	0.0364	EDG1	0.0176
PITX2	0.0058						
