# Supplementary material for: A Systems Biology-Based Gene Expression Classifier of Glioblastoma Predicts Survival with Solid Tumors
Source: PLoS One. 2009 Jul 17;4(7):e6274. doi: 10.1371/journal.pone.0006274 (PMC2707631; doi:10.1371/journal.pone.0006274)
Supplement: Table S7 — List of candidate survival-associated genes developed by method B from primary GBM data in UCLA. (0.03 MB PDF) [file pone.0006274.s013.pdf]

Table S7. List of candidate survival-associated genes developed by method B from primary GBM data in UCLA.

Gene Symbol	Score	Gene Symbol	Score	Gene Symbol	Score	Gene Symbol	Score
SSR1	0	ARGBP2	0.02	CART1	0	GPS2	0
MGC5306	0	CD19	0	GTF2I	0.03	FLJ21945	0.03
INSR	0.02	DNM1	0.01	MUC1	0.01	ADRBK1	0.05
LRP1	0	TYK2	0	CALM1	0	SHC3	0.02
PRKCA	0.05	JUNB	0	CBL	0.05	POU3F1	0.03
AR	0.04	JUND	0	GDNF	0.02	POU3F4	0.01
ATF2	0.01	TUBA1	0.03	CALR	0	SOD2	0
JUN	0.06	CREM	0.01	RXRB	0.01	HDHD3	0
MYC	0.01	RELA	0.05	CDK5	0	LMO4	0.02
YWHAE	0.01	TAF7L	0.01	DNMT2	0.05	MPHOSPH6	0
MCP	0.01	TITF1	0.02	POLA2	0.04	COX17	0
SYK	0.05	CDK5RAP3	0.01	TGFBR1	0	TMPRSS3	0.03
ZFP64	0.01	MAP3K8	0.02	NPPB	0	SKI	0.05
TERT	0	ATN1	0	BACH	0.03	SMG1	0
LARP1	0.04	KIAA0913	0.01	RGS3	0.05	PHYH	0
PTPN6	0.01	HRB	0.01	CASP3	0.02	ZBTB25	0.03
GNA13	0.04	CD79B	0	NOL3	0.04	SCAM-1	0.01
KLRA1	0.02	ITPR1	0.02	ETNK2	0	IMMT	0.02
LMO7	0.02	TOM1L1	0.03	PPL	0	IKBKE	0.01
PDLIM4	0	MAP2K6	0.05	TFAM	0.04	ANKRD12	0
CDH7	0	SRF	0.02	RAD23A	0.01	CORO2B	0
MET	0.02	BID	0.03	EPHA7	0.01	PSG9	0.04
TSC2	0	SMARCA4	0	KCNJ4	0.03	TFE3	0.02
SRRM2	0	SMARCC1	0	S100B	0.01	CEBPG	0.03
BIRC4	0.04	ELK1	0	MDFI	0.05	UBB	0.04
ATF1	0.03	GAB1	0.01	ZNF646	0.03	NRGN	0
CREBBP	0.05	CRKL	0	WNT1	0.01	PLCG1	0
RBPSUH	0.04	PTPRC	0.04	CHD8	0	CNOT7	0.01
SMAD4	0	BCL6	0.02	FAM60A	0.02	EFEMP2	0
HCK	0.02	MAPK1	0.01	LRPAP1	0.02	GTPBP3	0
IL5RA	0.03	FGF2	0.04	MDK	0.04	ERBB4	0.04
VSNL1	0.02	CTDP1	0	GPRASP1	0.01	KRT18	0.05
CD40	0.05	MN1	0.03	RTN4	0.04	R3HDM	0.02
RLBP1	0.03						
