# Supplementary material for: A Systems Biology-Based Gene Expression Classifier of Glioblastoma Predicts Survival with Solid Tumors
Source: PLoS One. 2009 Jul 17;4(7):e6274. doi: 10.1371/journal.pone.0006274 (PMC2707631; doi:10.1371/journal.pone.0006274)
Supplement: Table S8 — List of candidate survival-associated genes developed by method B from primary GBM data in UCSF-1. (0.03 MB PDF) [file pone.0006274.s014.pdf]

Table S8. List of candidate survival-associated genes developed by method B from primary GBM data in UCSF-1.

Gene Symbol	Score	Gene Symbol	Score	Gene Symbol	Score	Gene Symbol	Score
CBX7	0.02	EPHB4	0.03	PTOV1	0.04	NCBP1	0.01
HCK	0.04	PECAM1	0.05	CHD8	0.2	RRM2	0.02
IGF1R	0.02	ERF	0.02	NID1	0.03	ICMT	0.02
IRAK1	0.02	RRAS	0.03	CHPF	0.01	CNN3	0.02
JUN	0.01	GRIN1	0	VDP	0.03	IFNAR1	0
PLCG1	0.03	CDK2	0	YKT6	0	BMP4	0
ROBO1	0.01	GSPT1	0.03	RGS16	0.05	ACTB	0.04
CCND2	0.02	SHH	0.05	RPS6KA5	0	CEBPG	0
MDM2	0.02	ATP1A3	0.05	GRAP2	0.2	IL13RA2	0
NDEL1	0.04	GDI1	0.01	ITGB2	0.01	NFKB2	0.01
CREBBP	0.05	VAMP2	0	SH3BP2	0	GLG1	0.04
STAT1	0.02	KIAA1128	0	SYK	0.05	UPF2	0
ACTA1	0.03	NFKBIE	0	EPB41L3	0.01	GPRASP1	0.04
VIM	0.02	SH3GL3	0.02	CR2	0.01	DOCK3	0
AR	0.02	CDC25B	0.05	LAMA4	0	ATP4B	0.02
EP300	0.02	IFRD1	0.04	S100A4	0.04	MAPK10	0.04
UBE2I	0.02	CHGB	0	CHEK1	0.03	MAP2K4	0
CAV2	0	NFE2	0.02	FASN	0	ZFYVE16	0
IL13	0.05	SCNN1G	0.05	RPL31	0.05	SAT	0.03
IRS1	0.04	HSPA4	0	SLC1A1	0.04	EIF2S2	0.02
NTRK1	0	SH3GL2	0.01	ZNF165	0.05	CAPZB	0.04
PIK3R1	0.04	CTDP1	0.02	MTMR9	0	RPA2	0.02
MAPK8	0.05	CUTL2	0	ZAP70	0.03	TK1	0.02
COL5A2	0	KIF5A	0.05	SMAD9	0.04	YLPM1	0
ATXN1	0	TP53BP2	0.01	TNNT1	0.05	CYCS	0.04
RAPGEF4	0.01	NCOA2	0	GRIA1	0	ITGA4	0.04
SPEN	0	GRM3	0.01	NUMA1	0	CKS2	0.03
TMEM23	0.04	FOSL2	0.05	GJA7	0	SPTAN1	0
BCR	0.01	CTCF	0.01	TAF13	0.04	GRIA2	0
ALOX12	0	FLNA	0.01	BUB1	0.04	PRKCB1	0.02
YES1	0.03	TRAF2	0.02	CDC20	0.04	FOXO1A	0
NRP1	0.01	FCER1G	0.01	ACTC	0.01	MYCBP2	0
NGFR	0.05	TIAM1	0.02	ZNF42	0	NEFL	0.05
JAK2	0.01	CHP	0.03	RHOQ	0.02	DAG1	0.04
ARHGDIB	0.03	MAPK8IP2	0.01	CALD1	0.02		
