# Supplementary material for: A Systems Biology-Based Gene Expression Classifier of Glioblastoma Predicts Survival with Solid Tumors
Source: PLoS One. 2009 Jul 17;4(7):e6274. doi: 10.1371/journal.pone.0006274 (PMC2707631; doi:10.1371/journal.pone.0006274)
Supplement: Table S9 — List of candidate survival-associated genes developed by method B from primary GBM data in MDA. (0.03 MB PDF) [file pone.0006274.s015.pdf]

Table S9. List of candidate survival-associated genes developed by method B from primary GBM data in MDA.

Gene Symbol	Score	Gene Symbol	Score	Gene Symbol	Score	Gene Symbol	Score
EPN1	0	NFKBIA	0	ITGB2	0	TAF9L	0.05
DTX2	0.05	RB1	0.01	SUV39H1	0.03	GRB7	0.01
MTF2	0.05	XPO1	0.02	LRP2	0.01	CAV3	0
ASCC2	0.02	FLT3	0.01	SFRS2	0.11	ARRB1	0.02
EMD	0.01	SCNN1A	0.02	SFRS6	0	PPFIBP2	0
PCNA	0.01	RFC5	0	SMARCA3	0.03	FYB	0.02
TMPO	0.03	RPA2	0	MDC1	0.02	SFN	0.12
LYN	0	DYRK1A	0.01	CLTC	0	CSPG2	0.01
CBX8	0	PTN	0.01	POU3F4	0.04	HLA-DPA1	0.05
CDK2	0	RAD51C	0	DHX9	0.03	POLA	0
PTHLH	0	CD33	0.01	NR3C1	0.03	BID	0.05
COIL	0.02	HLA-A	0.04	NFKB1	0.02	IL1B	0.01
KIAA0980	0	RARB	0.01	SI	0.07	CLU	0.02
RSRC1	0.05	POLR2D	0.01	RUNX2	0.05	SCN5A	0.01
CAV1	0.04	DPPA4	0.01	ACTB	0.1	TIMELESS	0.02
PLCG2	0.02	GMFB	0.05	LAPTM5	0.02	POMT2	0
MYB	0.05	MYC	0.01	INCENP	0.03	IL8RB	0.03
E2F1	0	SREBF1	0.03	CBX5	0	PEG3	0.01
DNAJA3	0.02	KIF15	0.03	KIAA0179	0.01	PRKX	0.02
C20orf12	0.01	TLR2	0.01	MEIS1	0.07	APOE	0.03
CTNNB1	0	IRF4	0.02	PBX3	0.01	RHOH	0.03
MSH6	0	ZNF451	0.04	MSH2	0.01	DOK5	0.01
HSPD1	0.05	C1orf149	0	BTG3	0.03	DNMT3A	0
RAD51	0.02	PAWR	0	FLJ20364	0.01	HCK	0.02
CDC25A	0.05	TAC3	0.02	CTNND1	0.02	PRKD1	0.04
SMAD5	0.05	NCOR2	0.02	RAD1	0.07	MAP3K14	0
MCM2	0.03	CSF1R	0.02	SLD5	0	HLA-B	0.03
CCHCR1	0.02	NFKBIB	0.03	TRAF5	0.01	CSF3R	0.01
SKP2	0	RNPS1	0	STMN1	0.02	LCP2	0
NIF3L1	0.02	KRT18	0.02	SON	0.07	ACVR2A	0.01
FUSIP1	0.03	CDC6	0.04	INPP4A	0.05	ATF4	0.01
MYCBP	0.2	TP73	0.03	TNFRSF11A	0	MCM3	0.01
PIK3R3	0	KRT15	0.01	KRT6B	0.01		
