# Supplementary material for: A Systems Biology-Based Gene Expression Classifier of Glioblastoma Predicts Survival with Solid Tumors
Source: PLoS One. 2009 Jul 17;4(7):e6274. doi: 10.1371/journal.pone.0006274 (PMC2707631; doi:10.1371/journal.pone.0006274)
Supplement: Table S10 — List of prognostic genes developed by method B from primary GBM data in UCLA, UCSF-1, and MDA. (0.01 MB PDF) [file pone.0006274.s016.pdf]

Table S10. List of prognostic genes developed by method B from primary GBM data in UCLA, UCSF-1, and MDA.

**Description**	**Gene Symbol**	**Entrez ID**
BH3 interacting domain death agonist	BID	637
cyclin-dependent kinase 2	CDK2	1017
replication protein A2, 32kDa	RPA2	6118
CTD phosphatase, subunit 1	CTDP1	9150
phospholipase C, gamma 1 (formerly subtype 148)	PLCG1	5335
v-jun sarcoma virus 17 oncogene homolog (avian)	JUN	3725
