# Supplementary material for: A Systems Biology-Based Gene Expression Classifier of Glioblastoma Predicts Survival with Solid Tumors
Source: PLoS One. 2009 Jul 17;4(7):e6274. doi: 10.1371/journal.pone.0006274 (PMC2707631; doi:10.1371/journal.pone.0006274)
Supplement: Table S11 — List of candidate survival-associated genes developed by method C from primary GBM data in UCLA. (0.01 MB PDF) [file pone.0006274.s017.pdf]

Table S11. List of candidate survival-associated genes developed by method C from primary GBM data in UCLA.

Entrez ID	Gene Symbol	Entrez ID	Gene Symbol	Entrez ID	Gene Symbol	Entrez ID	Gene Symbol
4493	MT1E	2184	FAH	56255	DJ971N18.2	3423	IDS
4885	NPTX2	10530	RIG	23367	LARP1	5577	PRKAR2B
55268	ECHDC2	27295	PDLIM3	117178	SSX2IP	8836	GGH
2013	EMP2	9071	CLDN10	8871	SYNJ2	25891	DKFZP586H2123
6284	S100A13	104	ADARB1	6169	RPL38	8496	PPFIBP1
4499	MT1K	5634	PRPS2	28231	SLCO4A1	25907	RIS1
8991	SELENBP1	4495	MT1G	93664	CADPS2	8507	ENC1
1635	DCTD	25876	C20orf28	51477	ISYNA1	10076	PTPRU
29015	SLC43A3	4599	MX1	10581	IFITM2	1432	MAPK14
8572	PDLIM4	2157	F8	79022	MGC5576	9488	PIGB
64114	PP1201	30008	EFEMP2	55337	FLJ11286	90627	STARD13
3488	IGFBP5	79871	C1orf82	4192	MDK	54588	MXRA7
1534	CYB561	307	ANXA4	2192	FBLN1	25830	SULT4A1
51226	COPZ2	23633	KPNA6	25972	UNC50	64782	ISG20L1
2766	GMPR	10404	PGCP	6508	SLC4A3	655	BMP7
57212	KIAA0495	7360	UGP2	23215	BAT2D1	7130	TNFAIP6
51673	CGI-38	57030	SLC17A7	114884	OSBPL10	1039	CDR2
1307	COL16A1	51299	NRN1	11277	TREX1	81031	SLC2A10
3223	HOXC6	10410	IFITM3	760	CA2	23245	ASTN2
4494	MT1F	3708	ITPR1	79778	MICAL-L2	56339	METTL3
8996	NOL3	3939	LDHA	7037	TFRC	657	BMPR1A
6817	SULT1A1	7039	TGFA	8553	BHLHB2	7832	BTG2
3418	IDH2	622	BDH	873	CBR1	5587	PRKD1
10398	MYL9	4900	NRGN	32	ACACB	57820	CCNB1IP1
9609	RAB36	4502	MT2A	1346	COX7A1	637	BID
5919	RARRES2	56683	C21orf59	284119	PTRF	57117	PHF22
55742	PARVA	23075	SWAP70	6616	SNAP25	94134	ARHGAP12
4061	LY6E	54882	ANKHD1	6620	SNCB	114088	TRIM9
9645	MICAL2	10190	TXNDC9	9208	LRRFIP1	23127	GLT25D2
1499	CTNNB1	54969	FLJ20534	5327	PLAT	7570	ZNF22
9104	RGN	5999	RGS4	8819	SAP30	4862	NPAS2
4826	NNAT	55008	HERC6	3598	IL13RA2	5372	PMM1
55295	FLJ11078	6385	SDC4	23531	MMD	8566	PDXK
9956	HS3ST2	2249	FGF4	54897	CASZ1	283638	KIAA0284
5322	PLA2G5	24139	EML2	11000	SLC27A3	6622	SNCA
7164	TPD52L1	10602	CDC42EP3	22977	AKR7A3	3094	HINT1
10397	NDRG1	5471	PPAT	10505	SEMA4F	10391	CORO2B
7026	NR2F2	1525	CXADR	112950	MED8	1728	NQO1
6990	TCTE1L	51176	LEF1	7286	TUFT1	7277	TUBA1
56675	NRIP3	445	ASS	4496	MT1H	23181	C21orf106
80036	TRPM3	5805	PTS	10993	SDS	1174	AP1S1
6799	SULT1A2	64285	RHBDF1	55884	WSB2	10745	PHTF1
3964	LGALS8	1548	CYP2A6	91289	BC002942	79717	PPCS
6285	S100B						
