# Supplementary material for: A Systems Biology-Based Gene Expression Classifier of Glioblastoma Predicts Survival with Solid Tumors
Source: PLoS One. 2009 Jul 17;4(7):e6274. doi: 10.1371/journal.pone.0006274 (PMC2707631; doi:10.1371/journal.pone.0006274)
Supplement: Table S12 — List of candidate survival-associated genes developed by method C from primary GBM data in UCSF-1. (0.03 MB PDF) [file pone.0006274.s018.pdf]

Table S12. List of candidate survival-associated genes developed by method C from primary GBM data in UCSF-1.

Entrez ID	Gene Symbol	Entrez ID	Gene Symbol	Entrez ID	Gene Symbol	Entrez ID	Gene Symbol
23480	SEC61G	5631	PRPS1	23466	CBX6	119	ADD2
4057	LTF	871	SERPINH1	5297	PIK4CA	10529	NEBL
4060	LUM	3725	JUN	10396	ATP8A1	6363	CCL19
1278	COL1A2	3028	HADH2	58473	PLEKHB1	54453	RIN2
1281	COL3A1	3842	TNPO1	6844	VAMP2	2746	GLUD1
4837	NNMT	991	CDC20	147179	WIRE	9806	SPOCK2
10135	PBEF1	9689	BZW1	11215	AKAP11	29	ABR
4223	MEOX2	55816	DOK5	283638	KIAA0284	8514	KCNAB2
1116	CHI3L1	7431	VIM	1584	CYP11B1	26106	DKFZP586K1520
3485	IGFBP2	3308	HSPA4	80303	EFHD1	2220	FCN2
1268	CNR1	2787	GNG5	5730	PTGDS	7102	TSPAN7
2152	F3	389	RHOC	8525	DGKZ	801	CALM1
6648	SOD2	5686	PSMA5	23710	GABARAPL1	9173	IL1RL1
2014	EMP3	8754	ADAM9	140609	NEK7	8888	MCM3AP
5947	RBP1	8813	DPM1	770	CA11	23112	TNRC6B
7422	VEGF	25932	CLIC4	4601	MXI1	6720	SREBF1
2171	FABP5	6929	TCF3	599	BCL2L2	2492	FSHR
358	AQP1	10434	LYPLA1	9150	CTDP1	6122	RPL3
7045	TGFBI	22856	CHSY1	23158	KIAA0882	9284	NPIP
1282	COL4A1	8407	TAGLN2	5860	QDPR	3047	HBG1
10630	PDPN	7153	TOP2A	84162	KIAA1109	9804	TOMM20
3371	TNC	420676	C7orf24	3705	ITPK1	10314	LANCL1
1290	COL5A2	10949	HNRPA0	815	CAMK2A	3207	HOXA11
2173	FABP7	1017	CDK2	23164	M-RIP	10409	BASP1
2335	FN1	80317	ZNF306	333	APLP1	782	CACNB1
5806	PTX3	7298	TYMS	11202	KLK8	22924	MAPRE3
2633	GBP1	1647	GADD45A	23048	FNBP1	56311	ANKRD7
5359	PLSCR1	1019	CDK4	339287	LOC339287	6385	SDC4
5054	SERPINE1	5236	PGM1	8427	ZNF282	6900	CNTN2
3486	IGFBP3	10956	OS9	23387	KIAA0999	5870	RAB6A
3956	LGALS1	8970	HIST1H2BJ	26999	CYFIP2	5299	SERPINA2
3491	CYR61	3336	HSPE1	219654	C10orf56	8927	BSN
3821	KLRC1	10920	COPS8	527	ATP6V0C	23241	PACS2
3823	KLRC3	10130	PDIA6	4714	NDUFB8	1408	CRY2
6616	SNAP25	182	JAG1	9728	KIAA0256	7741	ZNF187
4900	NRGN	1031	CDKN2C	6340	SCNN1G	29907	SNX15
8537	BCAS1	86	ACTL6A	23558	WBP2	6407	SEMG2
7368	UGT8	1000	CDH2	246308	KLHL9	2113	ETS1
288	ANK3	7965	JTV1	2550	GABBR1	5578	PRKCA
4747	NEFL	5928	RBBP4	8036	SHOC2	9693	RAPGEF2
6812	STXBP1	4811	NID1	2194	FASN	23405	DICER1
5178	PEG3	2902	GRIN1	6709	SPTAN1	217	ALDH2
2891	GRIA2	23077	MYCBP2	9497	SLC4A7	5510	PPP1R7
11069	RAPGEF4	120	ADD3	9959	USP12	9612	NCOR2
9568	GPR51	84193	C14orf154	23177	KIAA0582	9478	CABP1
5354	PLP1	347	APOD	3812	KIR3DL2	5064	PALM
6252	RTN1	23345	SYNE1	1395	CRHR2	818	CAMK2G
7037	TFRC	7381	UQCRB	6638	SNRPN	3223	HOXC6
8905	AP1S2	4130	MAP1A	10360	NPM3	5663	PSEN1
7018	TF	23136	EPB41L3	5261	PHKG2	56650	C3orf4
8848	TSC22D1						
