# Supplementary material for: A Systems Biology-Based Gene Expression Classifier of Glioblastoma Predicts Survival with Solid Tumors
Source: PLoS One. 2009 Jul 17;4(7):e6274. doi: 10.1371/journal.pone.0006274 (PMC2707631; doi:10.1371/journal.pone.0006274)
Supplement: Table S13 — List of candidate survival-associated genes developed by method C from primary GBM data in MDA. (0.03 MB PDF) [file pone.0006274.s019.pdf]

Table S13. List of candidate survival-associated genes developed by method C from primary GBM data in MDA.

EntrezID	GeneSymbol	EntrezID	GeneSymbol	EntrezID	GeneSymbol	EntrezID	GeneSymbol
1017	CDK2	7112	TMPO	51363	GALNAC4S-6ST	6603	SMARCD2
2013	EMP2	6839	SUV39H1	718	C3	83955	FKSG17
4172	MCM3	7153	TOP2A	10858	CYP46A1	8125	ANP32A
5074	PAWR	9833	MELK	11118	BTN3A2	30	ACAA1
79000	C1orf135	29092	HSPC157	7879	RAB7	6678	SPARC
5947	RBP1	9918	CNAP1	51347	TAOK3	84939	MUM1
10127	ZNF263	1968	EIF2S3	26020	LRP10	23780	APOL2
8914	TIMELESS	10682	EBP	1153	CIRBP	9957	HS3ST1
23306	KIAA0286	10615	SPAG5	5141	PDE4A	23466	CBX6
10950	BTG3	6118	RPA2	195	AHNAK	23180	RAFTLIN
5422	POLA	8480	RAE1	2124	EVI2B	3122	HLA-DRA
5985	RFC5	4678	NASP	2209	FCGR1A	1778	DNCH1
9768	KIAA0101	1524	CX3CR1	402055	LOC402055	2357	FPR1
2618	GART	773	CACNA1A	10150	MBNL2	55901	THSD1
4171	MCM2	8623	ASMTL	5788	PTPRC	770	CA11
56938	ARNTL2	2123	EVI2A	10284	SAP18	222070	LOC222070
79135	MGC4825	4067	LYN	11031	RAB31	9805	SCRN1
51203	NUSAP1	9993	DGCR2	6319	SCD	23621	BACE1
79979	CXorf34	1462	CSPG2	5742	PTGS1	83660	TLN2
5810	RAD1	3119	HLA-DQB1	23091	KIAA0853	2891	GRIA2
22981	KIAA0980	7043	TGFB3	6404	SELPLG	6196	RPS6KA2
79022	MGC5576	50610	C22orf9	214	ALCAM	64108	IFRG28
29980	DONSON	1727	CYB5R3	7805	LAPTM5	10384	BTN3A3
94431	LOC94431	23273	KIAA0367	56034	PDGFC	719	C3AR1
10745	PHTF1	10875	FGL2	9882	TBC1D4	6036	RNASE2
2956	MSH6	7078	TIMP3	64231	MS4A6A	240	ALOX5
10204	NUTF2	6767	ST13	274	BIN1	94134	ARHGAP12
90861	C16orf34	3916	LAMP1	7305	TYROBP	7101	NR2E1
2528	FUT6	23492	CBX7	10964	IFI44L	8376	IFIT3
5433	POLR2D	4792	NFKBIA	2033	EP300	9848	MFAP3L
55355	DKFZp762E1312	1436	CSF1R	64798	DEPDC6	6503	SLA
55220	FLJ10748	3113	HLA-DPA1	5341	PLEK	3394	IRF8
7752	ZNF200	972	CD74	4973	OLR1	963	CD53
55268	ECHDC2	8881	CDC16	8082	SSPN	26011	ODZ4
29893	TBPIP	8611	PPAP2A	7070	THY1	57406	ABHD6
54908	FLJ20364	3115	HLA-DPB1	10979	PLEKHC1	8031	NCOA4
83461	CDCA3	4134	MAP4	65983	NS3TP2	28996	HIPK2
4090	SMAD5	8635	RNASET2	55273	FLJ10970	29082	CHMP4A
94104	C21orf66	6480	ST6GAL1	8717	TRADD	9770	RASSF2
1869	E2F1	351	APP	713	C1QB	6642	SNX1
10681	GNB5	4332	MNDA	2209	FCGR1A	10766	TOB2
983	CDC2	115207	KCTD12	11240	PADI2	824	CAPN2
5928	RBBP4	9414	TJP2	23258	RAB6IP1	23235	SNF1LK2
10051	SMC4L1	6348	CCL3	9935	MAFB	9445	ITM2B
5557	PRIM1	1410	CRYAB	6518	SLC2A5	23114	NFASC
80222	TARSL1	57817	HAMP	11078	HRIHFB2122	3108	HLA-DMA
52	ACP1	23136	EPB41L3	24144	TFIP11	55701	FLJ10357
92	ACVR2A	23317	DNAJC13	1496	CTNNA2	6604	SMARCD3
115353	LRRC42	199	AIF1	6001	RGS10	4329	ALDH6A1
5427	POLE2	83937	RASSF4	23034	SAMD4	1734	DIO2
11120	BTN2A1	50863	HNT	55303	GIMAP4	3118	HLA-DQA2
8473	OGT	1230	CCR1	3433	IFIT2	10656	KHDRBS3
6790	STK6	3689	ITGB2	23643	LY96	397	ARHGDIB
5889	RAD51C	7462	LAT2	221981	LOC221981	1837	DTNA
10921	RNPS1	9945	GFPT2	7070	THY1	6347	CCL2
24137	KIF4A	558	AXL	85477	SCIN	1191	CLU
81893	LAT1-3TM	3123	HLA-DRB1	8439	NSMAF	51816	CECR1
4173	MCM4	29760	BLNK	6376	CX3CL1	114088	TRIM9
1058	CENPA	6351	CCL4	9388	LIPG	66008	ALS2CR3
6418	SET	64581	CLEC7A	8404	SPARCL1	1312	COMT
6491	SIL	3109	HLA-DMB	2037	EPB41L2	3117	HLA-DQA1
79183	C20orf121	10384	BTN3A3	5654	PRSS11	53346	TM6SF1
1503	CTPS	26018	LRIG1	3782	KCNN3	11151	CORO1A
10229	COQ7	942	CD86	9450	LY86	2207	FCER1G
9735	KNTC1	1500	CTNND1	79682	MLF1IP	124152	MGC35048
4666	NACA	113	ADCY7	25941	C18orf10		
