# Supplementary material for: A Systems Biology-Based Gene Expression Classifier of Glioblastoma Predicts Survival with Solid Tumors
Source: PLoS One. 2009 Jul 17;4(7):e6274. doi: 10.1371/journal.pone.0006274 (PMC2707631; doi:10.1371/journal.pone.0006274)
Supplement: Table S14 — List of prognostic genes developed by method C from primary GBM data in UCLA, UCSF-1, and MDA. (0.01 MB PDF) [file pone.0006274.s020.pdf]

Table S14. List of prognostic genes developed by method C from primary GBM data in UCLA, UCSF-1, and MDA.

Description	Gene Symbol	Entrez ID
epithelial membrane protein 2	EMP2	2013
putative homeodomain transcription factor 1	PHTF1	10745
hypothetical protein MGC5576	MGC5576	79022
Rho GTPase activating protein 12	ARHGAP12	94134
transferrin receptor (p90, CD71)	TFRC	7037
retinol binding protein 1, cellular	RBP1	5947
glutamate receptor, ionotropic, AMPA 2	GRIA2	2891
topoisomerase (DNA) II alpha 170kDa	TOP2A	7153
cyclin-dependent kinase 2	CDK2	1017
retinoblastoma binding protein 4	RBBP4	5928
erythrocyte membrane protein band 4.1-like 3	EPB41L3	23136
