# Supplementary material for: A Systems Biology-Based Gene Expression Classifier of Glioblastoma Predicts Survival with Solid Tumors
Source: PLoS One. 2009 Jul 17;4(7):e6274. doi: 10.1371/journal.pone.0006274 (PMC2707631; doi:10.1371/journal.pone.0006274)
Supplement: Table S15 — Multivariate cox regression analysis of previously reported prognostic genes for glioma in training and validation cohorts of glioma. (0.01 MB PDF) [file pone.0006274.s021.pdf]

Table S15. Multivariate cox regression analysis of previously reported prognostic genes for glioma in training and validation cohorts of glioma.

			35 genes		44 genes		47 genes	
Glioma	Cohort	Covariate	P value	HR(CI95%)	P value	HR(CI95%)	P value	HR(CI95%)
GBM	UCLA	cluster	0.775	1.09 (0.59-2.01)	0.236	1.32 (0.83-2.11)	0.275	1.62 (0.68-3.85)
		age	<0.0001	1.06 (1.03-1.10)	0.01	1.05 (1.01-1.09)	<0.0001	1.06 (1.03-1.10)
	UCSF-1	cluster	0.006	1.77 (1.18-2.65)	0.011	1.47 (1.09-1.98)	0.001	4.34 (1.80-10.46)
	MDA	cluster	0.825	1.04 (0.71-1.54)	0.115	1.23 (0.95-1.95)	0.122	1.64 (0.89-3.08)
		age	0.769	1.01 (0.98-1.03)	0.768	1.01 (0.97-1.03)	0.46	1.01 (0.98-1.04)
	CMBC	cluster	0.005	2.17 (1.27-3.70)	0.013	1.61 (1.11-2.34)	0.046	2.50 (1.01-6.17)
	UCSF-2	cluster	0.035	2.18 (1.06-4.51)	0.004	3.13 (1.44-6.80)	0.021	4.46(1.26-15.82)
		age	0.092	1.04 (0.99-1.09)	0.021	1.07 (1.01-1.13)	0.048	1.06 (1.00-1.11)
HGG(Grade III&GBM)	UCLA	cluster	0.142	1.34 (0.91-1.98)	0.035	1.41 (1.02-1.93)	0.564	1.22 (0.62-2.42)
		grade	0.017	2.78 (1.20-6.43)	0.022	2.62 (1.15-5.99)	0.01	3.06 (1.30-7.22)
		age	0.227	1.01 (0.99-1.04)	0.245	1.01 (0.99-1.04)	0.169	1.02 (0.99-1.04)
	MDA	cluster	0.274	1.24(0.84-1.82)	0.009	1.48 (1.11-1.99)	0.603	1.21 (0.60-2.44)
		grade	0.051	2.23 (1.00-5.01)	0.056	2.11 (0.98-4.52)	0.021	2.49 (1.15-5.39)
		age	0.227	1.02(0.99-1.04)	0.39	1.01 (0.99-1.04)	0.234	1.02 (0.99-1.04)
	CMBC	cluster	0.045	1.69 (1.01-2.81)	0.002	1.93 (1.28-2.91)	0.017	2.57 (1.17-5.62)
		grade	0.177	1.80 (0.77-4.23)	0.234	1.64 (0.73-3.67)	0.156	1.82 (0.80-4.17)

The direction of the hazard ratio are as follows: cluster, the shorter versus longer survival group; grade, GBM versus Grade III; age, older versus younger.
