# Supplementary material for: A Systems Biology-Based Gene Expression Classifier of Glioblastoma Predicts Survival with Solid Tumors
Source: PLoS One. 2009 Jul 17;4(7):e6274. doi: 10.1371/journal.pone.0006274 (PMC2707631; doi:10.1371/journal.pone.0006274)
Supplement: Table S16 — Multivariate cox regression analysis of previously reported prognostic genes for glioma in training and validation cohorts of other tumors. (0.01 MB PDF) [file pone.0006274.s022.pdf]

Table S16. Multivariate cox regression analysis of previously reported prognostic genes for glioma in training and validation cohorts of other tumors.

			35 genes		44 genes		47 genes	
Tumor type	Cohort	Covariate	P value	HR (CI95%)	P value	HR (CI95%)	P value	HR (CI95%)
Breast	GIS	cluster	0.141	1.35 (0.91-2.01)	0.16	1.17 (0.94-1.45)	0.666	1.16 (0.60-2.25)
		grade	0.002	1.93 (1.28-2.91)	0.02	1.72 (1.09-2.73)	0.001	2.09 (1.37-3.19)
		age	0.965	1.00 (0.98-1.02)	0.925	1.01 (0.98-1.02)	0.997	1.00 (0.98-1.02)
	CRCM	cluster	<0.0001	2.34 (1.59-3.44)	0.003	1.63 (1.18-2.25)	<0.0001	5.36 (2.60-11.09)
		grade	<0.0001	3.75 (2.09-6.73)	<0.0001	2.93 (1.69-5.07)	<0.0001	4.18 (2.28-7.66)
		age	0.916	1.01 (0.97-1.04)	0.974	1.00 (0.97-1.03)	0.731	1.00 (0.96-1.03)
	SUSM	cluster	0.077	1.37 (0.99-1.90)	0.089	1.25 (0.97-1.62)	0.293	1.29 (0.81-2.05)
		grade	<0.0001	2.42 (1.70-3.46)	<0.0001	2.28 (1.57-3.32)	<0.0001	2.48 (1.72-3.56)
		age	0.022	0.96 (0.92-0.99)	0.019	0.96 (0.91-0.99)	0.022	0.96 (0.92-0.99)
	NCI	cluster	0.656	1.09 (0.74-1.62)	0.001	1.59 (1.20-2.11)	0.099	1.87(0.89-3.91)
	EMC	cluster	0.036	1.34 (1.02-1.75)	0.062	1.16 (0.99-1.36)	0.339	1.24 (0.80-1.91)
Lung	DFCI	cluster	0.276	1.24 (0.84-1.83)	0.14	1.28 (0.92-1.79)	0.86	1.07 (0.52-2.20)
		stage	0.099	1.96 (0.88-4.37)	0.072	2.14 (0.93-4.88)	0.133	1.85(0.83-4.12)
		age	0.104	1.03 (0.99-1.07)	0.203	1.03 (0.99-1.07)	0.091	1.03 (1.00-1.08)
	PCH	cluster	0.092	1.59 (0.93-2.72)	0.126	1.35 (0.92-1.99)	0.564	1.28 (0.56-2.92)
		age	0.262	1.02 (0.98-1.06)	0.216	1.03 (0.98-1.07)	0.346	1.02 (0.98-1.06)
	CAN/DF	cluster	0.854	1.06 (0.59-1.88)	0.349	1.15 (0.86-1.52)	0.942	1.03 (0.50-2.12)
		stage	0.003	2.82 (1.43-5.54)	0.004	2.71 (1.37-5.53)	0.003	2.82 (1.44-5.55)
		age	0.002	1.07 (1.03-1.12)	0.001	1.07 (1.03-1.12)	0.002	1.07 (1.03-1.12)
	MSK	cluster	0.019	2.06 (1.13-3.77)	0.301	1.23 (0.83-1.84)	0.325	1.55 (0.65-3.70)
		stage	0.342	1.56 (0.62-3.92)	0.187	1.84 (0.74-4.54)	0.253	1.71 (0.68-4.30)
		age	0.759	1.01 (0.96-1.06)	0.388	1.02 (0.97-1.07)	0.454	1.02 (0.97-1.07)
	UM-HLM	cluster	0.773	1.04 (0.82-1.13)	0.713	1.04 (0.83-1.31)	0.231	1.26 (0.86-1.85)
		stage	<0.0001	2.31 (1.56-3.42)	<0.0001	2.31 (1.56-3.42)	<0.0001	2.31 (1.57-3.42)
		age	0.002	1.03 (1.01-1.05)	0.002	1.02 (1.01-1.05)	0.003	1.03 (1.01-1.05)
Bladder	AUH	cluster	0.587	1.13 (0.73-1.76)	0.27	1.25 (0.84-1.84)	0.882	1.07 (0.46-2.46)
Ovarium	MNI	cluster	0.015	2.24 (1.17-4.29)	0.01	1.99 (1.18-3.36)	0.082	2.77 (0.88-8.70)
		age	0.044	1.07 (1.01-1.14)	0.059	1.06 (1.00-1.13)	0.066	1.06 (1.00-1.13)

The direction of the hazard ratio are as follows: cluster, the short-term versus long-term survival group; grade, lower versus higher; age, older versus younger.
