# Supplementary material for: A Systems Biology-Based Gene Expression Classifier of Glioblastoma Predicts Survival with Solid Tumors
Source: PLoS One. 2009 Jul 17;4(7):e6274. doi: 10.1371/journal.pone.0006274 (PMC2707631; doi:10.1371/journal.pone.0006274)
Supplement: Table S17 — 25 prognostic genes developed by method A (Cox regression) from primary GBM data in UCLA, UCSF-1, and MDA. 15 prognostic genes are overlapped genes between 25 prognostic genes and 23 prognostic genes (see Table 2 in the manuscript). (0.03 MB PDF) [file pone.0006274.s023.pdf]

Table S17. 25 prognostic genes developed by method A (Cox regression) from primary GBM data in UCLA, UCSF-1, and MDA. 15 prognostic genes are overlapped genes between 25 prognostic genes and 23 prognostic genes (see Table 2 in the manuscript).

**25 prognostic genes**		**15 prognostic genes**	
**Entrez ID**	**Gene Symbol**	**Entrez ID**	**Gene Symbol**
5880	RAC2	7283	TUBG1
3459	IFNGR1	7298	TYMS
5788	PTPRC	891	CCNB1
1432	MAPK14	9133	CCNB2
7409	VAV1	8317	CDC7
7283	TUBG1	7374	UNG
5599	MAPK8	6119	RPA3
613	BCR	990	CDC6
2353	FOS	6118	RPA2
7298	TYMS	6240	RRM1
891	CCNB1	1786	DNMT1
9168	TMSB10	9232	PTTG1
3939	LDHA	8836	GGH
8836	GGH	5880	RAC2
7374	UNG	3459	IFNGR1
6119	RPA3		
6240	RRM1		
8317	CDC7		
6118	RPA2		
990	CDC6		
9232	PTTG1		
9133	CCNB2		
5532	PPP3CB		
1786	DNMT1		
4067	LYN		
