# Supplementary material for: A Systems Biology-Based Gene Expression Classifier of Glioblastoma Predicts Survival with Solid Tumors
Source: PLoS One. 2009 Jul 17;4(7):e6274. doi: 10.1371/journal.pone.0006274 (PMC2707631; doi:10.1371/journal.pone.0006274)
Supplement: Table S18 — Multivariate cox regression analysis of 25 genes and 15 genes in training and validation cohorts of glioma. (0.01 MB PDF) [file pone.0006274.s024.pdf]

Table S18. Multivariate cox regression analysis of 25 genes and 15 genes in training and validation cohorts of glioma.

			**25 genes**		**15 genes**	
**Glioma**	**Cohort**	**Covariate**	**P value**	**HR (CI95%)**	**P value**	**HR (CI95%)**
GBM	UCLA	cluster	0.223	1.76 (0.71-4.36)	0.221	1.70 (0.73-4.00)
		age	0.001	1.06 (1.03-1.10)	<0.0001	1.06 (1.03-1.10)
	UCSF-1	cluster	0.002	3.67 (1.60-8.42)	0.002	3.67 (1.60-8.42)
	MDA	cluster	0.005	2.59 (1.33-5.02)	0.003	2.64 (1.38-5.04)
		age	0.921	1.00 (0.97-1.03)	0.913	1.00 (0.97-1.03)
	CMBC	cluster	0.011	5.87 (1.68-20.54)	0.054	2.34 (0.99-5.55)
	UCSF-2	cluster	0.022	4.06 (1.22-13.46)	0.037	4.24 (1.09-16.52)
		age	0.068	1.05 (1.00-1.10)	0.09	1.04 (0.99-1.09)
HGG (Grade III&GBM)	UCLA	cluster	0.524	1.25 (0.63-2.45)	0.284	1.38 (0.76-2.51)
		grade	0.003	3.89 (1.58-9.57)	0.004	3.19 (1.44-7.05)
		age	0.133	1.02 (1.00-1.04)	0.154	1.02 (0.99-1.04)
	MDA	cluster	0.001	2.47 (1.42-4.30)	0.007	2.08 (1.24-3.52)
		grade	0.006	2.81 (1.34-5.88)	0.022	2.39 (1.13-5.05)
		age	0.301	1.01 (0.99-1.04)	0.172	1.02 (0.99-1.04)
	CMBC	cluster	0.076	1.94 (0.93-4.04)	0.873	1.06 (0.51-2.19)
		grade	0.036	2.28 (1.06-4.93)	0.017	2.61 (1.19-5.76)

The direction of the hazard ratio are as follows: cluster, the short-term versus long-term survival group; grade, GBM versus Grade III; age, older versus younger.
