# Supplementary material for: A Systems Biology-Based Gene Expression Classifier of Glioblastoma Predicts Survival with Solid Tumors
Source: PLoS One. 2009 Jul 17;4(7):e6274. doi: 10.1371/journal.pone.0006274 (PMC2707631; doi:10.1371/journal.pone.0006274)
Supplement: Table S19 — Multivariate cox regression analysis of 25 genes and 15 genes in training and validation cohorts of other tumors. (0.01 MB PDF) [file pone.0006274.s025.pdf]

Table S19. Multivariate cox regression analysis of 25 genes and 15 genes in training and validation cohorts of other tumors.

			**25 genes**		**15 genes**	
**Tumor type**	**Cohort**	**Covariate**	**P value**	**HR (CI95%)**	**P value**	**HR (CI95%)**
Breast	GIS	cluster	0.086	1.66 (0.93-2.95)	0.223	1.45 (0.80-2.63)
		grade	0.007	1.80 (1.18-2.76)	0.001	2.03 (1.36-3.04)
		age	0.781	1.00 (0.98-1.02)	0.893	1.00 (0.98-1.02)
	CRCM	cluster	<0.0001	5.17 (2.63-10.17)	<0.0001	4.18 (2.20-7.97)
		grade	<0.0001	3.86 (2.18-6.85)	<0.0001	3.08 (1.76-5.40)
		age	0.87	1.00 (0.97-1.03)	0.642	0.99 (0.96-1.03)
	SUSM	cluster	0.029	1.78 (1.06-2.99)	0.405	1.23 (0.76-1.98)
		grade	0.001	2.21 (1.51-3.22)	<0.0001	2.51 (1.75-3.60)
		age	0.018	0.95 (0.92-0.99)	0.023	0.96 (0.92-0.99)
	NCI	cluster	0.04	2.22 (1.04-4.77)	0.046	2.08 (1.01-4.26)
	EMC	cluster	0.001	1.86 (1.27-2.73)	0.062	1.44 (0.98-2.12)
Lung	DFCI	cluster	0.027	2.25 (1.10-4.61)	0.019	2.37 (1.15-4.86)
		stage	0.147	1.81 (0.81-4.04)	0.187	1.72 (0.77-3.84)
		age	0.12	1.03 (0.99-1.07)	0.111	1.03 (0.99-1.07)
	PCH	cluster	0.448	1.39 (0.60-3.22)	0.079	2.49 (0.90-6.88)
		age	0.361	1.02 (0.98-1.06)	0.142	1.03 (0.99-1.08)
	CAN/DF	cluster	0.125	1.96 (0.83-4.65)	0.416	1.34 (0.66-2.70)
		stage	0.038	2.19 (1.05-4.59)	0.002	2.99 (1.50-5.94)
		age	0.001	1.08 (1.03-1.13)	0.001	1.07 (1.03-1.12)
	MSK	cluster	0.221	1.69 (0.73-3.94)	0.306	1.57 (0.66-3.70)
		stage	0.156	1.93 (0.78-4.78)	0.158	1.92 (0.78-4.76)
		age	0.394	1.02 (0.97-1.07)	0.303	1.03 (0.98-1.08)
	UM-HLM	cluster	0.485	1.15 (0.78-1.70)	0.598	1.11 (0.76-1.63)
		stage	<0.0001	2.21 (1.47-3.32)	<0.0001	2.25 (1.51-3.36)
		age	0.001	1.03 (1.01-1.05)	0.001	1.03 (1.01-1.05)
Bladder	AUH	cluster	0.049	2.22 (1.02-4.86)	0.072	2.05 (0.95-4.43)
Ovarium	MNI	cluster	0.419	1.59 (0.52-4.94)	0.553	1.43 (0.44-4.72)
		age	0.078	1.06 (0.99-1.12)	0.114	1.05 (1.00-1.12)

The direction of the hazard ratio are as follows: cluster, the short-term versus long-term survival group; grade, lower versus higher; age, older versus younger.
