# Supplementary material for: A Systems Biology-Based Gene Expression Classifier of Glioblastoma Predicts Survival with Solid Tumors
Source: PLoS One. 2009 Jul 17;4(7):e6274. doi: 10.1371/journal.pone.0006274 (PMC2707631; doi:10.1371/journal.pone.0006274)
Supplement: Table S21 — Multivariate cox regression analysis of prognostic gene sets in training and validation cohorts of glioma. (0.01 MB PDF) [file pone.0006274.s027.pdf]

Table S21. Multivariate cox regression analysis of prognostic gene sets in training and validation cohorts of glioma.

Glioma	Cohort	Covariate	P value	HR (CI95%)
GBM	UCLA	cluster	0.269	1.68 (0.67-4.24)
		age	0.001	1.06 (1.03-1.10)
	UCSF-1	cluster	0.198	1.58 (0.79-3.16)
	MDA	cluster	0.096	1.69 (0.91-3.11)
		age	0.653	1.01 (0.98-1.04)
	CMBC	cluster	0.088	2.29 (0.88-5.91)
	UCSF-2	cluster	0.021	4.46 (1.26-15.82)
		age	0.048	1.06 (1.00-1.11)
HGG (Glioma III&GBM)	UCLA	cluster	0.413	1.30 (0.69-2.43)
		grade	0.002	3.82 (1.65-8.85)
		age	0.106	1.02 (1.00-1.04)
	MDA	cluster	0.006	2.38 (1.28-4.39)
		grade	0.083	1.96 (0.92-4.21)
		age	0.282	1.01 (0.99-1.04)
	CMBC	cluster	0.245	1.55 (0.74-3.22)
		grade	0.009	2.71 (1.28-5.74)

The direction of the hazard ratio are as follows: cluster, the short-term versus long-term survival group; grade, GBM versus Grade III; age, older versus younger.
